# Causality-driven multivariate stock movement forecasting

**DOI:** 10.1371/journal.pone.0302197

**Published:** 2024-04-25

**Authors:** Abel Díaz Berenguer, Yifei Da, Matías Nicolás Bossa, Meshia Cédric Oveneke, Hichem Sahli

**Affiliations:** 1 Department of Electronics and Informatics (ETRO), Vrije Universiteit Brussel (VUB), Brussels, Belgium; 2 Fit-For-Purpose Technologies SRL, Brussels, Belgium; 3 Interuniversity Microelectronics Centre (IMEC), Heverlee, Belgium; University of Barcelona: Universitat de Barcelona, SPAIN

## Abstract

Our study aims to investigate the interdependence between international stock markets and sentiments from financial news in stock forecasting. We adopt the Temporal Fusion Transformers (TFT) to incorporate intra and inter-market correlations and the interaction between the information flow, i.e. causality, of financial news sentiment and the dynamics of the stock market. The current study distinguishes itself from existing research by adopting Dynamic Transfer Entropy (DTE) to establish an accurate information flow propagation between stock and sentiments. DTE has the advantage of providing time series that mine information flow propagation paths between certain parts of the time series, highlighting marginal events such as spikes or sudden jumps, which are crucial in financial time series. The proposed methodological approach involves the following elements: a FinBERT-based textual analysis of financial news articles to extract sentiment time series, the use of the Transfer Entropy and corresponding heat maps to analyze the net information flows, the calculation of the DTE time series, which are considered as co-occurring covariates of stock Price, and TFT-based stock forecasting. The Dow Jones Industrial Average index of 13 countries, along with daily financial news data obtained through the New York Times API, are used to demonstrate the validity and superiority of the proposed DTE-based causality method along with TFT for accurate stock Price and Return forecasting compared to state-of-the-art time series forecasting methods.

## 1 Introduction

The stock market plays an important role in a country’s economic and social organization. The International stock market indexes quote for several countries, including the stock daily open, high, low, close price, and volume among others. Stock Prices are essentially discrete-time series collected at regular intervals. Stock movement forecasting is a highly challenging task, as both micro and macroeconomic attributes and characteristics influence stock movement and political events and news [1]. The factors influencing stock market movements can be classified into *endogenous* and *exogenous* factors. Endogenous factors pertain to elements within the economic system, while exogenous factors originate from outside the economic system [2]. These factors contribute to the stock time series’ non-linearity, dynamic, and chaotic nature.

The past decades have witnessed a growing interest in stock movement prediction. Various statistical techniques available for time series regression analysis have been used, including multivariate adaptive regression splines (MARS) [3], autoregressive moving average (ARMA) [4], and Generalized AutoRegressive Conditional Heteroskedasticity (GARCH) [5]. In addition, academia has also used Neural Networks [6] to predict future stock prices through the historical time series price. Recent strides in deep learning algorithms have emerged as a focal point for researchers, offering promising avenues in stock movement prediction; see [7, 8] for surveys.

In recent deep learning literature, Transformers have been used to learn the correlations between endogenous and exogenous information to improve stock prediction. E.g. [9] utilized information from other stocks as auxiliary data and applied Transformer with Multi-Level contexts to enhance prediction performance. The work in [10] combined textual news and stock Price data from other stocks and used Transformer architecture [11] to summarize the significance of historical information in financial documents and stock Prices. They also used Transfer Entropy to detect causal relationships between stocks, which are then used as an attention mechanism to guide prediction.

In this paper, we follow the above trends to combine stock Prices along with financial news sentiments and the causal relationship between them and adopt the Temporal Fusion Transformers (TFT) [12], for stock Price and Return prediction.

Several recent papers have evlaluated the performance of TFT in economic forecasting. The authors of [13] considered that the fluctuations of one company’s stock Price probably affect companies that are in the same supply chain and suggested using TFT for training with other companies’ stock data to increase the accuracy of predicting a particular stock. TFT achieved the lowest errors compared to LSTM. More recently, the authors of [14] adopted TFT to predict the Chinese macroeconomic system’s performance, including macroeconomy, industries, services, and 70 subsectors indicators. For each indicator, they applied TFT, with as explanatory variables (observed inputs) the gross final product (GFP), productive investment, net exports, and intermediate goods, and as known inputs, they used the month of the time point to be forecast and the corresponding order of the last week within the month. Their experiments also used static covariates, which included the industry attribute of each forecast indicator. The authors compared TFT to LSTM and reached the same conclusions as in [13]. For analyzing Gross domestic product (GDP), Laborda and his colleagues [15] utilized TFT for the joint GDP forecasting of 25 OECD countries at different time horizons. They also evaluated the relative importance of the explanatory variables. They found that, among others, the CRB Raw Industrial Spot Index and the World Trade Indicator are good predictors of the OECD indicator.

In this work, we consider the causality between stocks and sentiments. However, different from the previous works using the correlation between news text and financial time series and Transfer Entropy from one stock to another as in [10], in our approach, we make use of Yao’s [16] Dynamic Transfer Entropy (DTE) to obtain a set of time series that reveals the transmission path of information among financial news sentiments and stock Price fluctuations to trace the specific time of causal changes between financial stocks and sentiments. The Dow Jones Industrial Average (DJIA) index of 13 countries and daily financial news data obtained through the New York Times (NYT) API demonstrate the proposed DTE-based’s validity and superiority. For accurate stock Price and Return forecasting, we adopt TFT to capture the complex temporal correlations between stock covariates and the multivariate correlations among causal changes between different time series. We compared TFT to the most recent state-of-the-art forecasting models that accept as inputs multivariate time series and showed outstanding performance on several forecasting tasks with varied architecture and modelling techniques, such that we cover various models for a comprehensive assessment.

## 2 Related works

Stock forecasting encompasses a wide range of themes, including stock prediction models, sentiment analysis in finance, causal detection, and the study of information flow. Given the vastness of this literature, conducting a comprehensive review of all previous methods is beyond the scope of this paper. Instead, we focus on presenting the most relevant and closely related works that align with our proposed approach.

We first introduce the studies of information flow analysis between financial markets and financial markets and sentiment, summarising the use of Granger Causality [17], and Shannon Transfer Entropy [18] in this context. We then provide recent research on stock movement prediction. As summarized in Table 1, we categorize stock movement prediction methods into a) those based on individual historical stock, b) those using historical stock and additional textual information, and c) methods combining historical stock movement from different countries/companies and d) methods combining historical stock movements and additional information such as news articles.

**Table 1:** Categories of stock forecasting models.

Category	References
Individual Stock Prediction	[19–21]
Stock Prediction Incorporating Text Information	[22, 23]
Stock Prediction Incorporating News Sentiment	[24–26]
Correlated Stock Prediction with Text Information	[9, 10]

### Causality between financial markets and sentiments

It is generally acknowledged that news events can substantially impact the short-term direction of stock Prices. Granger Causality [17], and Shannon Transfer Entropy [18] have been widely used in the finance field for causal analysis to quantify the extent of information transmission between markets and market and news sentiments time series. In the following, we review some of these works.

In [27], the authors used Shannon Transfer Entropy to quantify information flows between financial markets. They examined the importance of the credit default swap market relative to the corporate bond market for credit risk pricing. The work in [28] investigated the relations between Twitter and financial markets. Granger Causality analysis of the sentiment tweets with various stock Returns has shown that for many companies, there is a statistically significant causality between stock and the sentiments driven by tweets. The study in [29] used news articles from the Common Crawl News to measure the impact of the news sentiment on stocks in the S&P 500 index. Using a dataset from 3 January 2018 to 27 February 2020, they computed the Shannon Transfer Entropy between hourly Sentiment Score differences and hourly price returns for each company. They found evidence of statistically significant transfer of information on the intra-day level over the analysis period. The authors of [30] analysed the intensity with which negative and positive news affects stock Prices. Using Shannon Transfer Entropy, they compared the social sentiment movements with individual companies’ daily closing stock Prices. They confirmed that negative news affects stock Prices more than positive news. In [31], the authors studied information flow between the news sentiment and stock Price, emphasising the impact of news during the COVID-19 pandemic on the pharmaceutical sector. They applied Granger Causality, Shannon Transfer Entropy, and Rényi’s Transfer Entropy [32]. Their results suggest that the non-parametric Shannon and Rényi’s entropy approaches provide a superior tool for examining nonlinear causality, supplanting the Granger test, which is constrained to Gaussian time series with linear causation. They demonstrated that the information flows more strongly from sentiment to price, indicating that changes in sentiment have a notable influence on stock Prices.

Transfer entropy analysis has also been used for crypto markets. Keskin and Aste [33] investigated the effect of changes in social media sentiment on cryptocurrency returns. They considered Granger causality as well as Shannon Transfer Entropy. For the former, they calculated linear Transfer Entropy using ordinary least-squares regression, and for the latter, they used the multidimensional histogram approach for the density estimation [34]. They made use of the Effective Transfer Entropy (ETE) [35] to detect significant information transfer, with greater net information transfer from sentiment to price for ripple (XRP) and litecoin (LTC), and from price to sentiment for bitcoin (BTC) and ethereum (ETH).

In [16], Yao extended the use of ETE by proposing the Dynamic Transfer Entropy to estimate ETE with a sliding window methodology, the ETE at each time point is obtained via forward scrolling. Yao’s Dynamic Transfer Entropy (DTE) has been adopted in [36] to investigate the strength and direction of information flow among Economic Policy Uncertainty (EPU), investor sentiment, and the stock market. Their findings confirmed that Yao’s proposed method analysed better the lead-lag effect [37] between sentiment and stock Prices. Their results indicated that (a) EPU influenced investor sentiment, (b) the impact of sentiment on returns is non-significant, while the fluctuation in stock Price returns has a significant impact on investor sentiment, and (c) there is no direct information flow from EPU to the stock market.

### Individual stock prediction

Most individual stock movement prediction models are based on short-term memory units (LSTM). The authors of [19] compared LSTM and a 1-D CNN model to predict the stock closing price for 25 companies enlisted at the Bucharest Stock Exchange. The prediction in this study targeted the close price at time *t* + 1, taking into account its values from day *t*–*N*, where *N* is a given number of days back (window length). For their experiments, they set *N* = 30, the rolling window equal to 1 day, and the prediction length also to 1 day. The parameters of the LSTM were chosen manually, 3 LSTM layers, with 50 units in each layer and a dropout of 0.2. Adversarial training of an Attentive LSTM model has been proposed in [20]. The Attentive LSTM [38] introduced a temporal attention mechanism to select relevant encoder hidden states across all time steps. The authors argued that adversarial training accounts for the stochastic property of the stock market to learn the stock movement prediction model. To implement the adversarial training, they added perturbations on the last layer of the attentive LSTM, which is directly projected to the final prediction. The authors of [21] proposed a framework, denoted as CLSR, for stock movement prediction. CLRS consists of four main components: Historical Status, Hybrid Encoding Network, Contrastive Learning [39], and Supervised Learning. The Historical Status uses the Attentive LSTM presented above to capture the trend features of the stock Price series via the LSTM, and the attention mechanism weights the features at different moments. The Hybrid Coding Network has been designed to extract from the stock data via self-attention mechanism [40] global information and local information via Temporal Convolutional Networks [41]. For Contrastive Learning, they applied stochastic data augmentation to generate different views for intra-day information. They applied the model on 500 CSI-500 component stocks in the China A-share market from December 1, 2015, to December 1, 2019. The datasets consist of the opening price, high price, low price, closing price, and volume, which have been normalized. They demonstrated the effectiveness of the CLRS framework in terms of stock representations and stock movement prediction compared to the LSTM model of [20].

### Stock prediction incorporating text information

Some researchers have incorporated news articles and social media posts in their prediction models. As an example of this category of methods, we refer to the work of [22], who introduced StockNet, a deep generative model for stock movement prediction. StockNet comprises three primary components following a bottom-up fashion: (1) a Market Information Encoder (MIE) consisting of a forward GRU and a backward GRU that encodes tweets embeddings and stock Prices to temporal inputs; (2) a Variational Movement Decoder (VMD) that infers latent driven factor and decodes stock movements from the inputs. In their implementation, VMD adopts an RNN with a GRU cell to extract features and decode stock signals recurrently; and (3) an Attentive Temporal Auxiliary (ATA) that integrates temporal loss through an attention mechanism for model training. With the same purpose of exploiting text data, the authors of [23] proposed the Multi-head Attention Fusion Network (MAFN) for stock Price prediction. MAFN follows an encoder-decoder framework. The MAFN’s encoder consists of three layers: an Embedding Layer, a Multi-head Attentive Fusion Layer, and a Sequential Encoding Layer. The Embedding Layer embeds the texts for a given stock on a day. The Multi-head Attentive Fusion Layer projects the text embeddings into different semantic subspaces, each associated with a particular latent aspect-level representation. Using the attention mechanism, it then fuses multiple aspect-level representations for all the texts. Finally, the Sequential Encoding Layer feeds a sequence of the encoded text representations into an LSTM to obtain the corresponding hidden states. The MAFN’s decoder consists of three layers: Attentive Reading Layer, Sequential Decoding Layer, and Attentive Prediction Layer. The Attentive Reading Layer aggregates the hidden states from the encoder using the traditional attention mechanism for each decoding step. The Sequential Decoding Layer is an LSTM that takes the context vectors and stock price as input to extract features sequentially. Finally, the Attentive Prediction Layer, which focuses on temporal attention to discriminate the importance of temporal features, provides the final prediction of the stock closing price.

### Stock prediction incorporating news sentiment

Methods in this category adopt a two-step approach. The first step is dedicated to extracting sentiment from financial texts or tweets. The second step uses the extracted Sentiment Score with stock information for stock movement prediction. The work of [24] considered news articles and the Taiwan PTT forum discussions collected from the Internet by crawler, and daily transaction information of individual stocks from the Taiwan Stock Exchange Corporation, which includes opening price, closing price, highest price, lowest price, and transaction volume. They used a BERT [42] model for sentiment analysis to determine the polarity of an input sentence. For a given stock and date, they estimate the probability of positive/negative articles or posts, defined as the ratio of the number of positive articles over the total number of articles of that date. Then, they combine, from the past 20 days, the four-dimensional data of sentiments from news articles and forum posts (2× positive and negative probabilities) with the Min-Max Normalized five stock information. The obtained multivariate time series (9-dimentional) is used as input for an LSTM prediction model to forecast the stock Prices for individual stocks. The authors concluded that the sentiments implicit in news and forums play an important role in the stock market, affecting the changes in stock Prices. A comparative study conducted by [25] explored the use of BERT [42] and FinBERT [43], a specialized language model based on BERT for sentiment analysis on financial texts. The authors focused their study on AMAZON stock for 5 months. For the purpose of stock prediction, they evaluated three models: (1) a single-layer LSTM model using solely historical stock Prices, (2) a single-layer LSTM model using multivariate inputs, including Sentiment Scores extracted using BERT and historical stock Prices, and (3) a single-layer LSTM model using multivariate inputs, incorporating Sentiment Scores extracted using FinBERT and historical stock Prices. The authors concluded that Sentiment Scores extracted using FinBERT outperformed those obtained using BERT for stock prediction. In [26], the authors proposed an LSTM-based Weighted and Categorized News Stock prediction model (WCN-LSTM), incorporating news categories with their learned weights to predict stock trends. Their model combines 4 LSTM models, one for the Stock close price, volume, and technical indicators, one for Market-related news Sentiment Scores, one for Sector-related news Sentiment Scores, and one for Stock-related news Sentiment Scores; the outputs of the LSTM are concatenated and passed to a dense layer with a sigmoid activation to predict stock trends. For the sentiment analysis, they used sentiment lexicons and compared general sentiment lexicons such as VADER [44] and Harvard IV (HIV4) (https://inquirer.sites.fas.harvard.edu/homecat.htm), and Loughran–McDonald financial sentiment dictionary (LMD) [45] to estimate Sentiment Scores.

### Correlated stock prediction with text information

The last category of stock prediction methods combines historical stock movements from different countries/companies and text information from news articles. An attention-based model that exploits the correlations between stocks denoted as the Data-axis Transformer with Multi-Level contexts (DTML), has been proposed in [9]. DTML consists of three main modules: (1) An Attentive context generation module that captures the temporal correlations within the historical prices of each stock via attention LSTM that utilizes multivariate features of each day. Thus, they extract stock contexts from multivariate features by temporal attention. (2) A Multi-level context aggregation module to generate multi-level context vectors by combining local contexts generated from individual stocks and a global market context generated from historical index data. The combination is made using the dot product between local and global context vectors; as a result, the global movement is incorporated in the individual contexts, affecting the inter-stock correlations. (3) A Data-axis self-attention module to learn the dynamic stock correlations using a transformer encoder to correlate the generated multi-level contexts by attention scores. The obtained attention maps are incorporated in the final predictions as correlations that change dynamically over time. The authors demonstrated that DTML achieved state-of-the-art accuracy on six datasets for stock movement prediction.

Considering the causality between stocks combined with financial text, Luo *et al*. [10] proposed the Causality-guided Multi-memory Interaction Network (CMIN). CMIN consists of three modules. The first module is the Feature Embedding module, which includes two encoders, one for embedding the textual information and another for embedding the price time series. Additionally, a global *causality matrix* has been introduced to capture the asymmetric correlations to guide the calculation of attention weights in the second module. The *causality matrix* is the Transfer Entropy matrix generated for each monitoring window by calculating the Shannon Transfer Entropy [32] between all stocks using their historical closing prices. The second module, denoted Multi-Memory Networks, consists of (i) a Text Memory Network composed of a multi-head attention layer followed by a GRU cell unit that updates the current hidden state into the next hidden state and outputs it to the next layer as the new continuous representation, and (ii) a Stock Correlation Memory Network employed to dynamically identify stock relationships and update the continuous representation of stock correlations. The last module, the Multi-Directional Interaction module, allows textual and causality information to reinforce each other for better prediction performance. The authors compared CMIN against 4 state-of-art models for binary classification, namely, the Attentive LSTM model of [38], the adversarial Attentive LSTM (Adv-LSTM) model of [20], the StockNet of [22], and the DTML of [9].

### Contributions

The above works demonstrated the benefits of using transformers and/or causality matrices to correlate stocks from different countries and transformers to capture the influence of temporal stock movements and textual information. In this work, we follow the same research trends for stock Price and Return forecasting and propose a methodology that combines stocks from different countries and financial news sentiments. We use Yao’s [16] Dynamic Transfer Entropy to obtain a set of time series that reveals the transmission path of information among financial news sentiments and stock Price fluctuations and trace the specific period of causal changes between financial stocks and sentiments, which are used as covariates for stock prediction using the TFT [12].

We adopted TFT as it considers various input variables and has shown outstanding performance on several forecasting tasks. The TFT introduces several novel architectures useful to our stock forecasting problem: (i) It encodes and selects valuable information from various heterogeneous covariates for forecasting. It considers a variable selection mechanism for each type of input. These mechanisms learn to weigh the importance of each input feature, such that the subsequent LSTM Sequence-to-Sequence layer will take as input the re-weighted sums of transformed continuous features inputs for each time step. (ii) It captures temporal dependencies at different time scales by combining the LSTM Sequence-to-Sequence to capture the short-term temporal dependence and a self-attention mechanism to capture the long-term time correlation in stock time series. Finally, (iii) TFT builds an explicit interpretation ability, which follows the trend of Explainable Artificial Intelligence (XAI) [46], it preserves interpretability by incorporating global, temporal dependency and events.

## 3 Stock market forecasting

**Fig 1:**
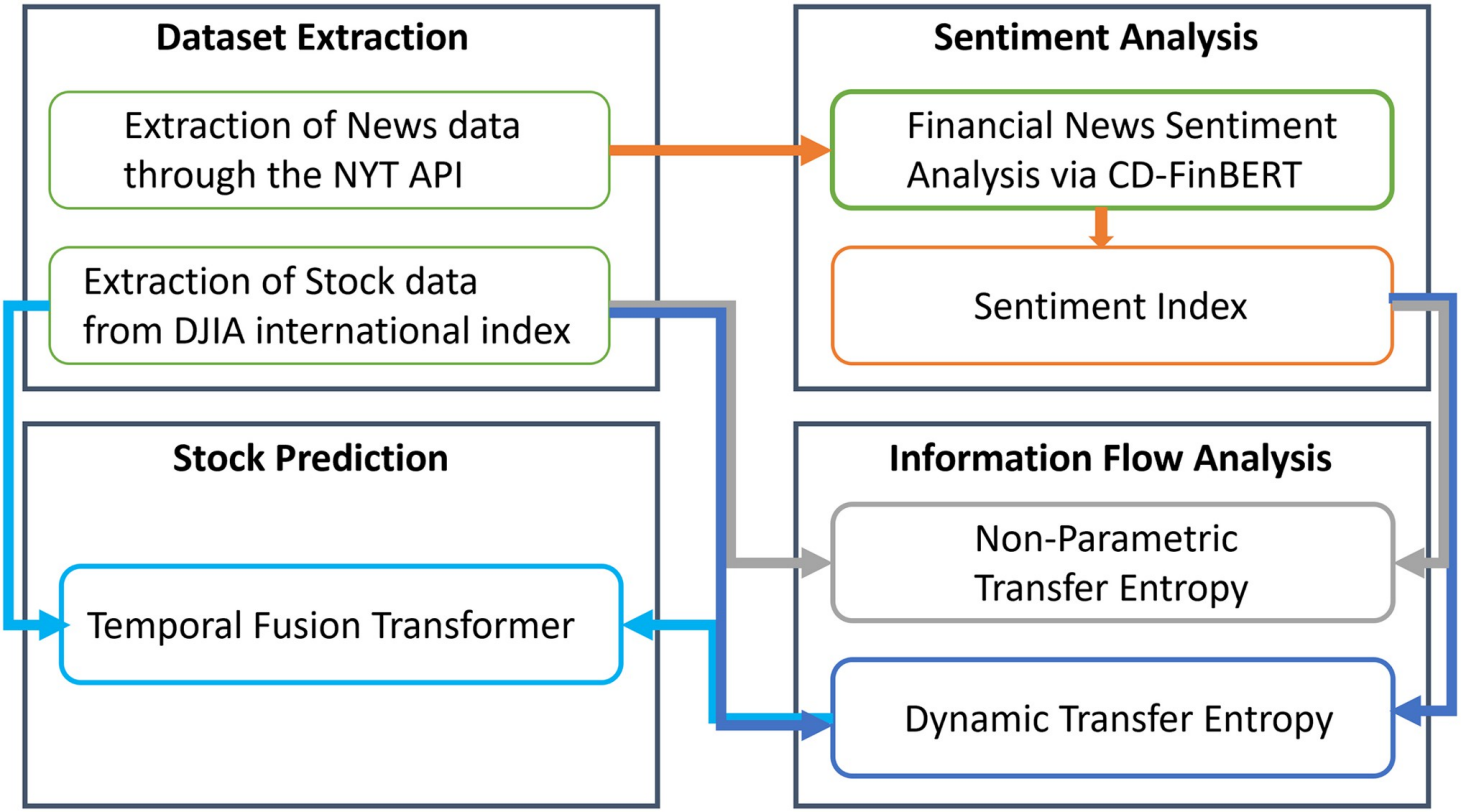
Schematic representation of our methodology. It consists of five stages: 1) Financial and News Dataset Extraction, 2) News Sentiment Analysis, 3) Transfer Entropy-based causal analysis to quantify causal inference between stock price/return and financial news sentiments, 4) Dynamic Transfer Entropy (DTE) to obtain time series that reveals the transmission path of information among financial news sentiments and stock price/return, and 5) Stock price/return prediction using as inputs time series of financial price/return and DTE.

A five-stage approach, as illustrated in Fig 1, is employed for this study. The initial stage entails data acquisition, gathering news articles and stock Prices for various countries (section 3.1: Datasets extraction). The collected financial news then undergoes the sentiment analysis module (section 3.2: Sentiment analysis). The subsequent step involves applying non-parametric Transfer Entropy for causal analysis to quantitatively assess the relationship and trace the information flow between stock Prices and news sentiment (section 3.3: Casual analysis). Then, we apply the Dynamic Transfer Entropy based on a sliding window analysis proposed by Yao [16] to obtain a time series that reveals the transmission path of information among financial news sentiments and stock Price fluctuations and trace the specific period of causal changes between financial stocks and sentiments (section 3.3.1: Dynamic transfer entropy). The obtained time series are considered as co-occurring covariates, which are used along with the stock Price and Return as inputs to the TFT [12] for stock Price and Return forecasting (section 3.4: Computational method for stock movement forecasting). In the following, we detail each of the stages.

### 3.1 Datasets extraction

The datasets utilized in our study comprise two main components: (i) financial data from the Dow Jones Industrial Average (DJIA) international index and (ii) text data sourced from the New York Times (NYT) financial news articles covering the period from March 02, 2020, to October 31, 2021, providing us with a comprehensive timeline, of 609 days, for our analysis.

#### Financial data

The DJIA international index holds significant importance as a financial index used to assess the economic status of a nation. Our selection encompasses 13 prominent indexes: Australia, Belgium, Canada, China, Denmark, Germany, Indonesia, Italy, Malaysia, Norway, Singapore, and South Korea. The choice of these countries is based on two considerations. Firstly, we prioritize nations with substantial financial magnitude, as their influence on other countries tends to be relatively stronger. Secondly, we exclude countries that have experienced prolonged market closures. By doing so, we ensure that the market opening days align closely among all selected countries, facilitating a fairer basis for comparison.

Typically, the Historical DJIA index for a specific country, company, or organization consists of seven columns of data: *Date*, *Price*, *Open*, *High*, *Low*, *Vol*, and *Change*. In this study, we precisely predict the *Price*, as it holds utmost importance in market analysis, and the *Return*. The closing price for each date forms the financial time series to be forecasted in our study. Apart from the daily stock Price, we also forecast the Return:

$$\begin{array}{c} r_{t}=\frac{p_{t+1}-p_{t}}{p_{t}} \end{array}$$ (1)

where *r*_*t*_ is the return at the date *t*, *p*_*t*+1_ and *p*_*t*_ are stock Prices at date *t* + 1 and *t*. This representation is used to measure stock movement in stock Exchanges. Both the Price, $P^{i}=\left\{p_{1}^{i},\cdots ,p_{T}^{i}\right\}$, and Return $R^{i}=\left\{r_{1}^{i},\cdots ,r_{T}^{i}\right\}$ time series of the considered countries, *i* ∈ [0, 12], are used as endogenous factors within this study.

#### Financial news data

**Fig 2:**
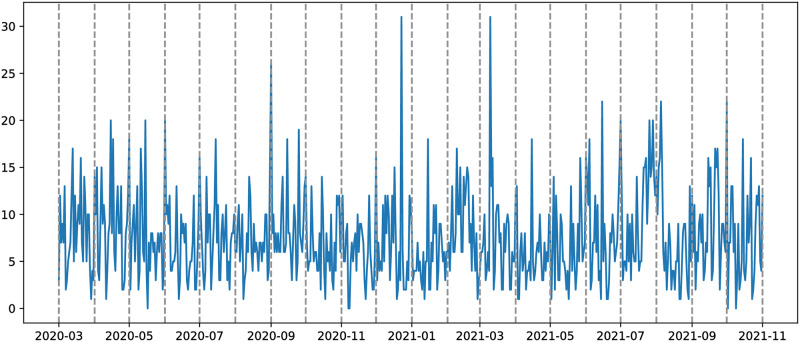
Daily news for Australia. The period covered from March 02, 2020, to October 31, 2021.

The daily financial news data is obtained through the New York Times (NYT) API (https://developer.nytimes.com/apis), which allows for the retrieval of relevant news articles based on specific keywords, such as country name and a designated period. For a given news article, the NYT API provides the following components: *abstract*, *web_url*, *snippet*, *lead_paragraph*, *print_section*, *print_page*, *source*, *multimedia*, *headline*, *keywords*, *pub_date*, *document_type*, *news_desk*, *section_name*, *byline*, and *type_of_material*. We analysed each of the components and realized that the *snippet* component contains the same information as the *abstract* component, and similarly, the *main* and *print_headline* components within the *headline* section share identical content, and the other components do not contain sentiment information. Hence, we consider only the *abstract*, *lead_paragraph*, and the *headline_main* components for the sentiment analysis as they contain most of the valuable news information. The *lead_paragraph* is chosen as it represents the main content of the news article. Table 2 summarises the statistics of the analyzed new papers per country, while Fig 2 visually depicts the daily news related to Australia.

**Table 2:** Maximum/Minimum/Average daily newspapers retrieved for the considered 13 countries. The period covered from March 02, 2020, to October 31, 2021.

	Max	Min	Avg
**Australia**	31	0	7.52
**Belgium**	14	0	2.00
**Canada**	32	0	8.09
**China**	74	1	19.30
**Denmark**	12	0	2.05
**Germany**	48	0	12.14
**Greece**	23	0	3.57
**Indonesia**	16	0	1.45
**Italy**	51	0	10.55
**Malaysia**	5	0	0.81
**Norway**	14	0	1.90
**Singapore**	21	0	1.66
**South Korea**	54	0	4.46

During the text pre-processing step, we observed that news articles are typically well-written and do not contain ambiguous words or misspellings. Therefore, our pre-processing involved only removing web addresses from the text.

### 3.2 Sentiment analysis

News on the economic state of a nation contains positive or negative sentiments depending on whether the information provided is favourable or unfavourable to the considered country. Cultural bias of financial analysts can affect stock analysis or recommendations in news articles. The cultural bias of financial analysts has been confirmed in a recent study of “Cultural Biases in Equity Analysis” by Pursiainen [47], who stated, “I find evidence that significant political events can introduce new cultural biases that are strong enough to affect stock recommendations”. Recognising that sentiment analysis models learn information from text corpora; they can pick up on and possibly amplify such biases present in the data. To lower country bias in sentiment analysis of financial news articles, we applied fine-tuning [48] to the 4 last layers of the FinBERT model [43] by imposing a fairness constraint [49].

For this purpose, we first build a counterfactual augmentation dataset using the subset “sentences_AllAgree” from the financial phrase-bank dataset [50]. The “sentences_AllAgree” consists of 2264 sentences, from which we selected the sentences that explicitly reference one of the considered countries. For each of the selected sentences, we created 12 counterfactual sentences by replacing the country name with the other 12 country names, obtaining 923 counterfactual sentence pairs. Then, we employed the counterfactual sentence pairs for fine-tuning the 4 last layers of the FinBERT model using the following loss function [49]:

$$\begin{array}{c} L\left(z\right)=L_{classification}\left(z\right)+\lambda .L_{fairness}\left(z,\tilde{z}\right) \end{array}$$ (2)

with *z* and $\tilde{z}$ two counterfactual sentences, $L_{classification}\left(.\right)$ the cross-entropy loss, λ a regularization parameter, and $L_{fairness}\left(.\right)$ the *sentiment regularization* fairness loss of [49]:

$$\begin{array}{c} L_{fairness}\left(z,\tilde{z}\right)=1-\frac{g^{T}\left(\bar{h}\left(z\right)\right)\cdot g\left(\bar{h}\left(\tilde{z}\right)\right)}{\left\|g\left(\bar{h}\left(z\right)\right)\|\|g\left(\bar{h}\left(\tilde{z}\right)\right)\right\|} \end{array}$$ (3)

where *g*(⋅) is the embedding from the last layer of the FinBert model and ∥ ⋅ ∥ is the natural norm.

For each news article, we use the fine-tuned FinBert model, *CD*_*FinBbert*, to generate Sentiment Scores in the range [−1, 1] for the *abstract*, *lead_paragraph*, and the *headline_main*, separately and then estimate the average as Sentiment Score for that specific news article. For a particular country, we estimate the average Sentiment Scores of all news articles of that day, denoted as *sc*_*t*_. In cases where no financial news articles are available for a particular day, we assume a Sentiment Score of 0, i.e., *neutral*. We obtain a time series of daily average Sentiment Scores for each considered country and the analysis period.

In our study, we also consider the Sentiment Index, *si*_*t*_, defined as:

$$\begin{array}{c} si_{t}=\frac{np_{t}-nn_{t}}{np_{t}+nn_{t}} \end{array}$$ (4)

here, *np*_*t*_ is the count of positive news articles on day *t*, and *nn*_*t*_ is the count of negative news articles on day *t*. News articles with a Sentiment Score *sc*_*t*_ > 0 are considered positive, and those with *sc*_*t*_ < 0 are counted as negative. If there is no news on that day, we assign the value 0 to the Sentiment Index of that day. Finally, for a given country *i*, we denote the sentiment time series as $Sc^{i}=\left\{sc_{1}^{i},\cdots ,sc_{T}^{i}\right\}$ and $Si^{i}=\left\{si_{1}^{i},\cdots ,si_{T}^{i}\right\}$, used in the next step of causal analysis as well as covariates for the stock prediction.

### 3.3 Causal analysis

Our study adds to the growing literature on causal analysis of news sentiment and financial stock in several ways. We first summarize the theory behind Transfer Entropy (TE) and the factors to be considered for its computation. We then investigate the information flow between the news sentiment and stock Price using TE. TE can identify linear and nonlinear interactions. It is also able to identify the directionality of the interaction. The TE analysis for this study was performed using a Python package (PyCausality) created by Zac Keskin and maintained on Keskin’s public GitHub (https://github.com/ZacKeskin/PyCausality).

Transfer Entropy [18] is based on Shannon’s entropy [51], which measures the amount of information in random processes. TE estimates the reduction of uncertainty of the observations of *Y* (target process), accounted by both the past observations of *X* (driver process) and the past observations of *Y*, compared to the reduction of uncertainty of the observations of *Y* accounted only by its past [18].

Two factors are essential for computing TE: defining the time series’ past observations and estimating the entropy. Selecting past observations is essential; a long past will lead to redundancy and increased computational requirements and a short past could lead to insufficient information. Considering the time lag *k* for past information, we can describe the information transfer from *X*_*t*−*k*_ to *Y*_*t*_ in terms of the following conditional mutual information:

$$\begin{array}{c} TE_{X\to Y}^{\left(k\right)}=H\left(Y_{t}|Y_{t-k}\right)-H\left(Y_{t}|X_{t-k},Y_{t-k}\right) \end{array}$$ (5)

This study adopts the kernel density estimator (KDE) [52] for estimating the probability density functions using Gaussian kernels.

#### 3.3.1 Dynamic transfer entropy

As described above, TE relies on non-parametric methods to estimate the probability distribution of the time series; this can introduce estimation errors, even in large samples. Marschinski & Kantz [35] proposed a measure denoted as Effective Transfer Entropy, which compares the calculated Transfer Entropy score with the average of a set of calculations over shuffled time series:

$$\begin{array}{c} ETE=TE-{\bar{TE}}_{shuffle} \end{array}$$ (6)

where ${\bar{TE}}_{shuffle}$ is the mean of the shuffled values. In this work, the 2 time series are shuffled together, and a 50 shuffling is adopted. By calculating the mean and standard deviation of the shuffled Transfer Entropy scores, one can estimate the significance of a causal result as:

$$\begin{array}{c} Z=\frac{TE-{\bar{TE}}_{shuffle}}{\sigma_{shuffle}} \end{array}$$ (7)

with *σ*_*shuffle*_ the standard deviation. The Z-score provides a distance, measured in terms of standard deviations, of the observed Transfer Entropy with respect to the expected value for non-causally related variables. Larger Z-scores imply a larger likelihood of causal relation. We use the Z-score to retain causality links because it is a robust statistical validation that depends on minimal assumptions. According to [16], assuming the distribution is close to Gaussian, a result with *Z* > 3 is roughly in the top 1% of results, comparable to a p-value significance testing with a p-value of 0.01. In this work, TE is used to quantitatively assess the relationship and trace the information flow between Prices/Return and Sentiment (section 4.1: Information flow analysis).

Yao [16] extended the use of ETE and Z-score and proposed the Dynamic Transfer Entropy for estimating ETE/Z-score with a sliding window methodology with TE at each time point obtained via forward scrolling. In this work, we use DTE (building upon the code kindly provided by Can-Zhong Yao [16]) to calculate causality time series that reveals the transmission path of information among financial news sentiments and stock Price fluctuations and trace the specific period of causal changes between financial stocks and sentiments. Moreover, the calculated DTE time series are used in this work as covariates for stock prediction (section 4.4: Price and return forecasting). To the best of our knowledge, this is the first study that uses Dynamic Transfer Entropy to predict stock Price and Return.

### 3.4 Computational method for stock movement forecasting

#### 3.4.1 Stock prediction problem definition

Let us assume a set of *C* aligned univariate time series denoted as $X={\left\{x_{1:T}^{i}\right\}}_{i=1}^{C}$, where the *i*^*th*^ time series is formally stated as $x_{1:T}^{i}=\left[x_{1}^{i},\cdots ,x_{T}^{i}\right]$, with $x_{t}^{i}\in R$ indicating the stock Price or Return value at time *t*, for instance Price, and *t* representing discrete time points equally distributed, say days. In addition, let $V={\left\{v_{1:T}^{i}\right\}}_{i=1}^{C}$ be a set of co-occurring or associate time-varying covariates where $v_{t}^{i}\in R^{d}$ denotes a vector of covariates at time *t*. Given, past observations of *X* and *V*, the objective of stock market prediction methods is to forecast unknown future univariate time series expressed as ${\hat{x}}_{T+1:T+n}^{i}=\left[{\hat{x}}_{T+1}^{i},\cdots ,{\hat{x}}_{T+n}^{i}\right]$, where ${\hat{x}}_{t+n}^{i}\in R$ indicates the forecasting value, and *n* expresses the discrete distance between the target forecasting and observed time points, thus delineating the future prediction horizon. Without loss of generalization such an objective can be formally expressed as follows:

$$\begin{array}{c} {\hat{x}}_{T+1:T+n}^{i}=F\left(X,V;\Theta \right) \end{array}$$ (8)

${\hat{x}}_{t+n}^{i}$ denotes a point estimate of ${\hat{x}}_{T+1:T+n}^{i}$, *F* represents a computational model for forecasting parameterized by Θ that denotes a set of learning parameters. One can notice from this equation that, while our goal is to predict ${\hat{x}}_{T+1:T+n}^{i}$ in the future forecasting horizon, the co-occurring covariates (i.e., *V*) are assumed to be known over the entire period, that is also in the future. Thus, following previous works [53, 54], we define the observation period as a training stage and assume that during inference in the prediction horizon, these associated covariates are given. In a real-world application, we can transfer the last observed covariates to the prediction horizon.

A significant aspect to consider in stock market forecasting concerns the co-occurring or associate covariates. Multiple factors, including the opinions of investors, the emotions of traders, the views of the public, and the news can influence the stock market [55]. Accordingly, this work focuses on the study of sentiment analysis and information flow underlying causality between news, sentiment, and the past stock market. The motivation behind this study is to leverage the benefits of combining domestic and foreign *endogenous* and *exogenous* data to forecast future domestic stock market trends.

#### 3.4.2 Time series processing. The rolling windows

Time series analysis often uses the rolling windows approach [56] because it provides a flexible manner to cope with time series data. This approach divides the time series into smaller, fixed-length windows to be used separately. In this manner, one can capture localized dynamics while retaining the temporal context, which helps identify patterns and fluctuations in the data over time at a local level. Hence, this approach is particularly appealing in forecasting problems.

Let us recall an *i*^*th*^ univariate time series $x_{1:T}^{i}=\left[x_{1}^{i},\cdots ,x_{T}^{i}\right]$, defined in Section 3.4.1. To apply the rolling windows approach, one defines a window size, say *w*, which determines the number of consecutive values to be included in each window. Hence, the number of windows, *τ*, can be calculated as *τ* = *T* − *w* + *m*, where *m* denotes the number of incrementing time points between successive rolling windows. This windowing process is repeated until one reaches the end of the time series, which leads to a transformed time series that involves multiple consecutive windows. Then, the first rolling window includes the time points from 1 through *w*; the second rolling window contains the time points from 1 + *m* through *w* + *m*, and so forth.

In this study, we look at multi-horizon stock forecasting requiring prediction for one week and then use the actual data from that week to forecast the subsequent week. We follow the multi-step forecasting with the walk-forward technique proposed in [57]. It operates by training the computational models with the records in the training dataset and then using the model to forecast the upcoming week’s records as the test dataset. In a practical scenario, when a week for which the last forecasting was made is over, its actual records are also included in the training dataset to forecast the upcoming week. This round of training forecasting is repeated recursively and so forth. Such a technique fits well for rolling windows as the windows can operate on a weekly basis. Once the model predicts, the window is shifted ahead, by the number of periods equal to test set to continue training [58]. The one-week-based multi-horizon prediction has also been found [59] to be the most pragmatic, considering that real-world applications mainly require forecasts up to one week. As a consequence, our rolling windows operate on a per-week basis.

To carry out stock market forecasting, we adopt the TFT [12] using as covariates the causal information flow involving financial news Sentiments and Price. We also compared TFT to different state-of-the-art computational methods for time series forecasting. We have selected the following computational models considering that they have shown outstanding performance on several forecasting tasks and, more importantly, that they vary in terms of architecture and modeling techniques, such that we cover a variety of models for a comprehensive assessment:

- Long Short-Term Memory (LSTM) [60] often used in the literature for stock Price prediction.
- Transformer [11] which has demonstrated its effectiveness for time-series forecasting. It captures contextual information and encode long-term dependencies through its self-attention mechanism.
- Probabilistic Forecasting with Autoregressive Recurrent Networks (DeepAR) [54] is a seminal work on time series forecasting, leveraging RNNs with a probabilistic approach. It incorporates a probabilistic approach by modeling the entire probability distribution of future predictions to capture uncertainty and generate stochastic forecasts. DeepAR is able to capture complex and group-dependent relationships by using covariates, and has demonstrated good performances in different application fields.
- Deep State Space (DSS) [53] model connects the perspectives of state-space models and RNNs into a method for multi-horizon forecasting. The DSS architecture consists of an RNN that processes the sequential input covariates into hidden states subsequently used to compute the parameters of a linear state-space model. The DSS has proven to achieve promising results in different application fields, it can also deliver interpretable predictions.

#### 3.4.3 Temporal Fusion Transformers (TFT) [12]

**Fig 3:**
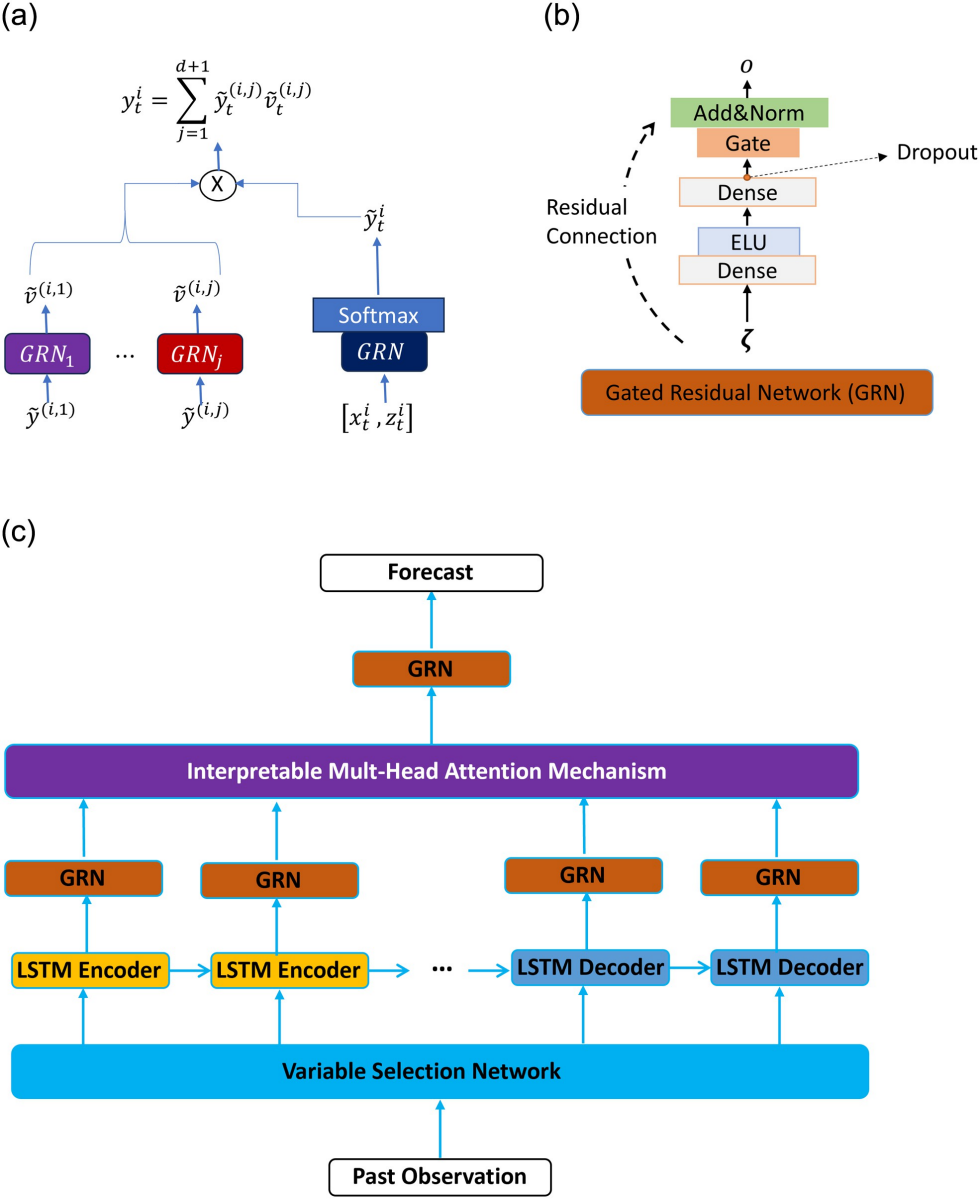
TFT architecture adapted from [12]. Variable Selection is used for selecting the most salient features based on the input. Gated Residual Network blocks enable information flow. Time-dependent processing is based on LSTMs for local processing, and multi-head attention for global processing. (**a**) Variable Selection Network (**b**) Gated Residual Network (**c**) Simplified TFT Architecture.

TFT is a model for multi-horizon time series forecasting. It combines the benefits of RNN and self-attention layers to capture short- and long-term temporal relationships. The two key components of TFT are its Gating Residual Network (GRN), allowing the model to adapt its depth and complexity to diverse data and scenarios and its Interpretable Attention blocks. Fig 3 illustrates the TFT architecture.

The GRN is a gating mechanism to tailor the model by applying non-linear transformation only when required according to the nature of the input covariates. The GRN consists of two stacked intermediate layers followed by gated linear units (GLU) [61] and normalization [62] layers. For hypothetical inputs, say *ζ*, the GRN is formally defined as follows [12]:

$$\begin{array}{c} l_{1}=\text{ELU}\left(W_{1}\zeta +b_{1}\right) \end{array}$$ (9)

$$\begin{array}{c} l_{2}=W_{2}l_{1}+b_{2} \end{array}$$ (10)

$$\begin{array}{c} \tilde{o}=\text{Sigmoid}\left(\left(W_{3}l_{2}+b_{3}\right)⊙\left(W_{4}l_{2}+b_{4}\right)\right) \end{array}$$ (11)

$$\begin{array}{c} o=\text{LayerNorm}(\zeta +\tilde{o}) \end{array}$$ (12)

where *l*_1_ and *l*_2_ are the outputs of the two intermediate layers with *W*_1_, *W*_2_, *b*_1_, and *b*_2_ their respective learnable parameters. ELU denotes the Exponential Linear Unit activation function [63]. The $\tilde{o}$ is the output of the GLU layer with learning parameters *W*_3_, *W*_4_, *b*_3_, and *b*_4_. LayerNorm is the normalization layer, and $o_{t}^{i}$ is the final outcome of the GRN. For simplicity and without loss of generalization, in the following we refer to the GRN as GRN(⋅; *θ*_*grn*_) and to the GLU (i.e., Eq 11) as GLU(⋅; *θ*_*glu*_), with *θ*_*grn*_ and *θ*_*glu*_ representing their corresponding set of learnable parameters. Although the TFT can employ GRN with a context vector to capture information from the non-temporal inputs, it is worth noticing that we do not use static inputs in this work.

The Interpretable Multi-Head Attention blocks help to capture long-term dependencies better. These blocks extend classical multi-head attention [11] with an additive aggregation of all heads to share their values. It enables each head to learn different patterns while remaining attentive to relevant features, formally as follows [12]:

$$\begin{array}{c} \text{InteMultiHeadAttention}\left(Q,K,V\right)=\tilde{H}VW^{h} \end{array}$$ (13)

$$\begin{array}{c} \tilde{H}=\{\frac{1}{H}\sum_{h=1}^{H}Attention\left(QW_{h}^{q},KW_{h}^{k}\right)\}VW^{v} \end{array}$$ (14)

$$\begin{array}{c} =\frac{1}{H}\sum_{h=1}^{H}Attention\left(QW_{h}^{q},KW_{h}^{k}\right)VW^{v} \end{array}$$ (15)

The temporal processing layers of the TFT first incorporate a data-driven Variable Selection Network to reduce the influence of irrelevant time-varying covariates. If necessary, the Variable Selection Network transforms the input covariates into a *d*-dimensional embedding to guarantee that skip connections match in the following layers. To this end, it employs the entity encoding [64] approach to attain linear layers capable of appropriately dealing with categorical and continuous covariates. Next, the Variable Selection Network also employs the GRN to learn the relevance of each input variable and incorporates a SoftMax activation to output a vector of weights that expresses the importance of corresponding inputs, say $x_{t}^{i}$ and $z_{t}^{i}$, as follows [12]:

$$\begin{array}{c} {\tilde{y}}_{t}^{i}=\text{SoftMax}(\text{GRN}\left(\left[x_{t}^{i},v_{t}^{i}\right];\theta_{grn}\right) \end{array}$$ (16)

$$\begin{array}{c} {\tilde{v}}_{t}^{\left(i,j\right)}={\text{GRN}}_{j}\left({\tilde{y}}^{\left(i,j\right)};\theta_{grn}\right) \end{array}$$ (17)

$$\begin{array}{c} y_{t}^{i}=\sum_{1}^{d+1}{\tilde{y}}_{t}^{\left(i,j\right)}{\tilde{v}}_{t}^{\left(i,j\right)} \end{array}$$ (18)

where [⋅] is a concatenation operator, the output ${\tilde{y}}_{t}^{i}\in R^{j}$, with *j* = *d* + 1, denotes the vector of weights to enable variable selection, ${\tilde{v}}^{\left(i,j\right)}$ is a feature map resulting from feeding each *j*^*th*^ input variable into an additional GRN_*j*_ operating per each *j*^*th*^ input, and $y_{t}^{i}$ is the weighted feature vector with the selected covariates.

TFT utilizes an encoder-decoder architecture for learning short- and long-term temporal relationships from observed temporal inputs. This encoder-decoder operates over the observation and forecasting periods to obtain the temporal features that serve as input to the decoder itself in the prediction horizon by generating forecasting sequentially. The encoder stack time-distributed Variable Selection Network to embed the most salient input covariates, followed by LSTM layers and gated skip connections, as follows [12]:

$$\begin{array}{c} h_{t}^{i}=\text{LSTM}\left(h_{t-1}^{i},y_{t}^{i};\theta_{lstm}\right) \end{array}$$ (19)

$$\begin{array}{c} {\tilde{m}}_{t}^{i}=\text{GLU}\left(h_{t}^{i};\theta_{grn}\right) \end{array}$$ (20)

$$\begin{array}{c} m_{t}^{i}=\text{LayerNorm}\left({\tilde{m}}_{t}^{i}+y_{t}^{i}\right) \end{array}$$ (21)

where $h_{t}^{i}$ is the encoder LSTM hidden state and $m_{t}^{i}$ denotes the encoded temporal features.

The resulting temporal features are fed to the decoder of TFT. This decoder uses a GRN-based enrichment layer followed by the Interpretable Multi-Head Attention blocks with masking to ensure attention only on preceding temporal features, and it operates over the observation and forecasting period. Next, it stacks a GLU layer, followed by layer normalization with skip connections. Finally, the decoder applies additional non-linear transformations by using another GRN, followed by GLU normalization layers, formally [12]:

$$\begin{array}{c} {\tilde{m}}_{t}^{i}=\text{GRN}\left(m_{t}^{i};\theta_{grn}\right) \end{array}$$ (22)

$$\begin{array}{c} j_{t}^{i}=\text{InteMultiHeadAttention}\left({\tilde{m}}_{t}^{i},{\tilde{m}}_{t}^{i},{\tilde{m}}_{t}^{i}\right) \end{array}$$ (23)

$$\begin{array}{c} {\tilde{j}}_{t}^{i}=\text{GLU}\left(j_{t}^{i};\theta_{grn}\right) \end{array}$$ (24)

$$\begin{array}{c} a_{t}^{i}=\text{LayerNorm}\left({\tilde{j}}_{t}^{i}+{\tilde{m}}_{t}^{i}\right) \end{array}$$ (25)

$$\begin{array}{c} r_{t}^{i}=\text{GRN}\left(a_{t}^{i};\theta_{grn}\right) \end{array}$$ (26)

$$\begin{array}{c} {\tilde{r}}_{t}^{i}=\text{GLU}\left(r_{t}^{i};\theta_{glu}\right) \end{array}$$ (27)

$$\begin{array}{c} {\hat{x}}_{t}^{i}=\text{LayerNorm}\left({\tilde{r}}_{t}^{i}+{\tilde{r}}_{t}^{i}\right) \end{array}$$ (28)

where ${\hat{x}}_{t}^{i}$ denotes the forecasting point estimate that can be only generated in the prediction horizon.

To generate the forecasting estimates during the prediction horizon, TFT employs a linear layer to transform the decoder output for providing multi-horizon forecasts by yielding prediction intervals on top of forecasting time points to output the 10^*th*^, 50^*th*^, and 90^*th*^ percentiles simultaneously [12]:

$$\begin{array}{c} {\hat{x}}_{t,p}^{i}=W_{p}{\hat{x}}_{t}^{i}+b_{p} \end{array}$$ (29)

where *W*_*p*_ and *b*_*p*_ are the corresponding linear operators per percentile.

Note that TFT allows three kinds of input types, namely static or time-invariant features, to provide additional information and context to the model, past-observed time-varying input and future-known time-varying input, such as calendar and other time-information. In this work, for a fair comparison with the other forecasting models, we considered only past observations of *X* and *V* as inputs to the TFT.

## 4 Experimental results

In this section, we discuss our experimental results. We start by analyzing the Information flow (i.e., the third stage of our methodology) between the Sentiment Index and the stock data. We then capture via DTE the dynamically evolving causal relationships between the Sentiment Index and stock data (i.e., the fourth stage of our methodology). For DTE estimation and all subsequent experiments, we fixed the sliding window size at 10, unless explicitly stated otherwise. Subsequently, we will discuss our stock market forecasting results using the Temporal Fusion Transformer (i.e., the fifth stage of our methodology) and compare these results to the stock market predictions of contemporary computational methods summarized in the previous section. Finally, we discuss the advantages of using the Temporal Fusion Transformer for deriving interpretable insights into stock forecasting, which are of utmost importance in real-world financial applications.

### 4.1 Information flow analysis

In the following, we report and discuss the TE analysis of the different combinations of the STock/Sentiment time series (Price&Sent.score, Return&Sent.score, Price&Sent.index, and Return&Sent.index). We chose a 1-day (i.e., *k* = 1) time lag for the four cases because longer lags showed weaker causal signals over this data.

**Fig 4:**
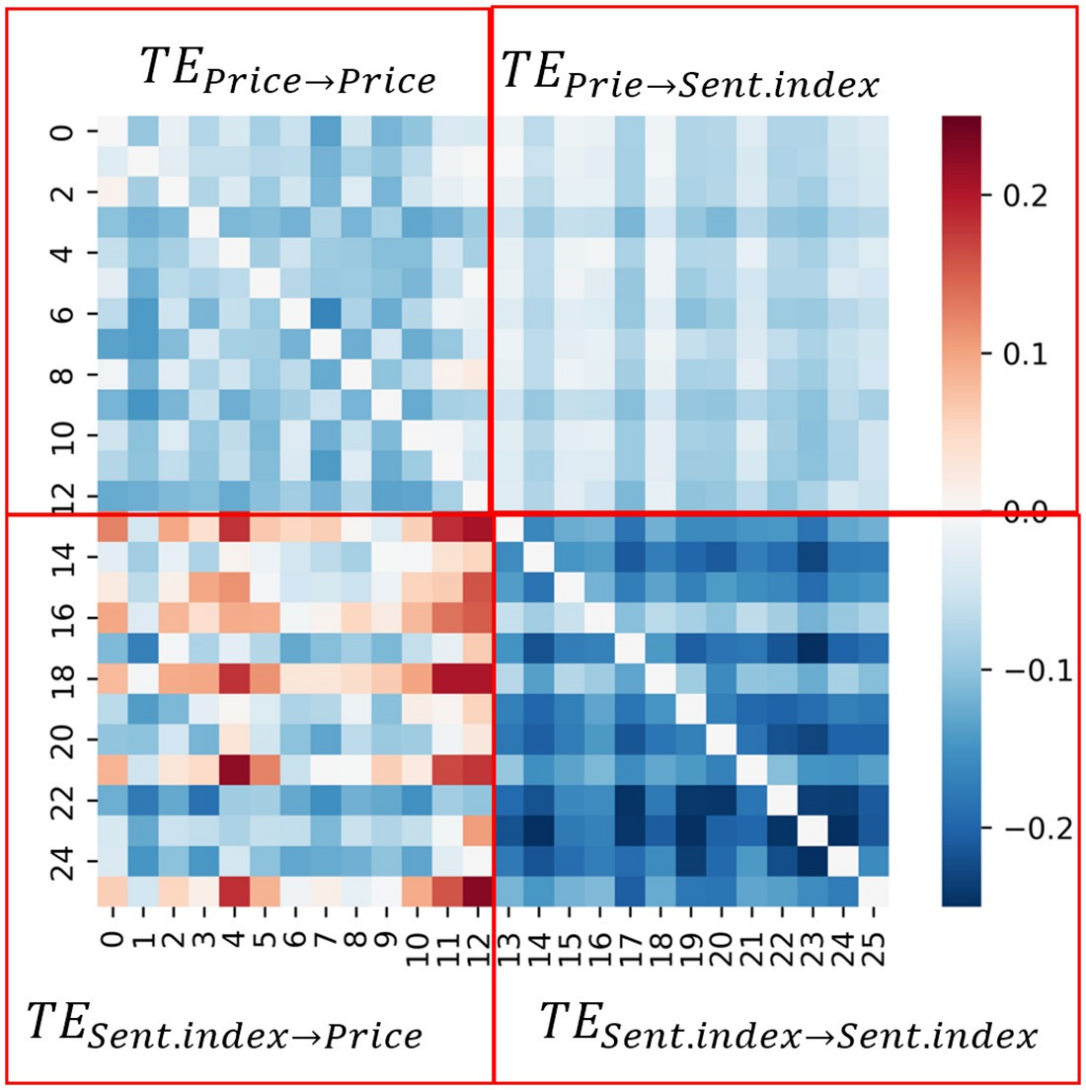
Illustration of the Causality Matrix. The top-left block is the information flow from Price to Price. The top-right block is the information flow from Price to Sentiment. The bottom-left block is the information flow from Sentiment to Price. The bottom-right block is the information flow from Sentiment to Sentiment.

For each country of the 13 countries analyzed in this work, we use the stock Price/Return and Sentiment Score/Index to calculate the Transfer Entropy between the time series and generate a Transfer Entropy Matrix called the Causality Matrix, formally denoted as *CM*. The obtained Causality Matrix (i.e, $CM\in R^{26\times 26}$) is illustrated in Fig 4 as Heat Maps.

The matrix *CM* illustrates the asymmetric flow of information from one stock to another and from sentiments to stocks. Each column, 0 ≤ *j* ≤ 12, in the Causality matrix *CM*, i.e., *CM*[:, *j*], quantifies the degree of information flow from other stocks to the stock of country *c* = *j*, as well as the flow from sentiment to it. The columns 13 ≤ *j* ≤ 25 represent the Transfer Entropy from the sentiment towards a country *c* = *j* − 13 to the stocks of the different countries and the sentiments towards them. We also calculated the Z-score Matrix using the same reasoning for the rows and columns.

**Fig 5:**
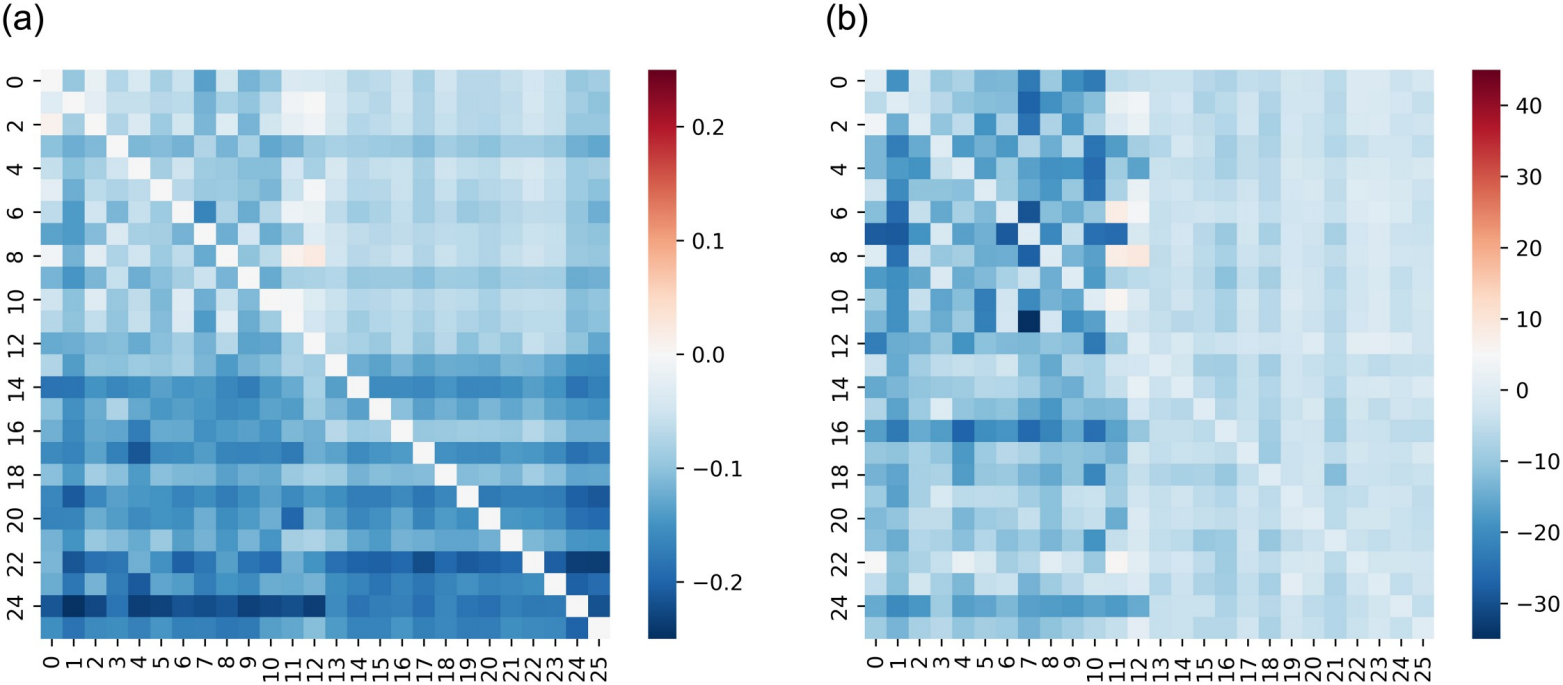
Price&Sent.score—Causality Matrix and Significance (Z-score) Matrix. No particular pattern indicating specific information flow. (**a**) TE (Return&Sent.score) (**b**) Significance (Return&Sent.score).

For the configuration Price&Sentiment Score, the Causality Matrix and the Significance (Z-scores) Matrix of Fig 5 do not exhibit a particular pattern indicating specific information flow.

**Fig 6:**
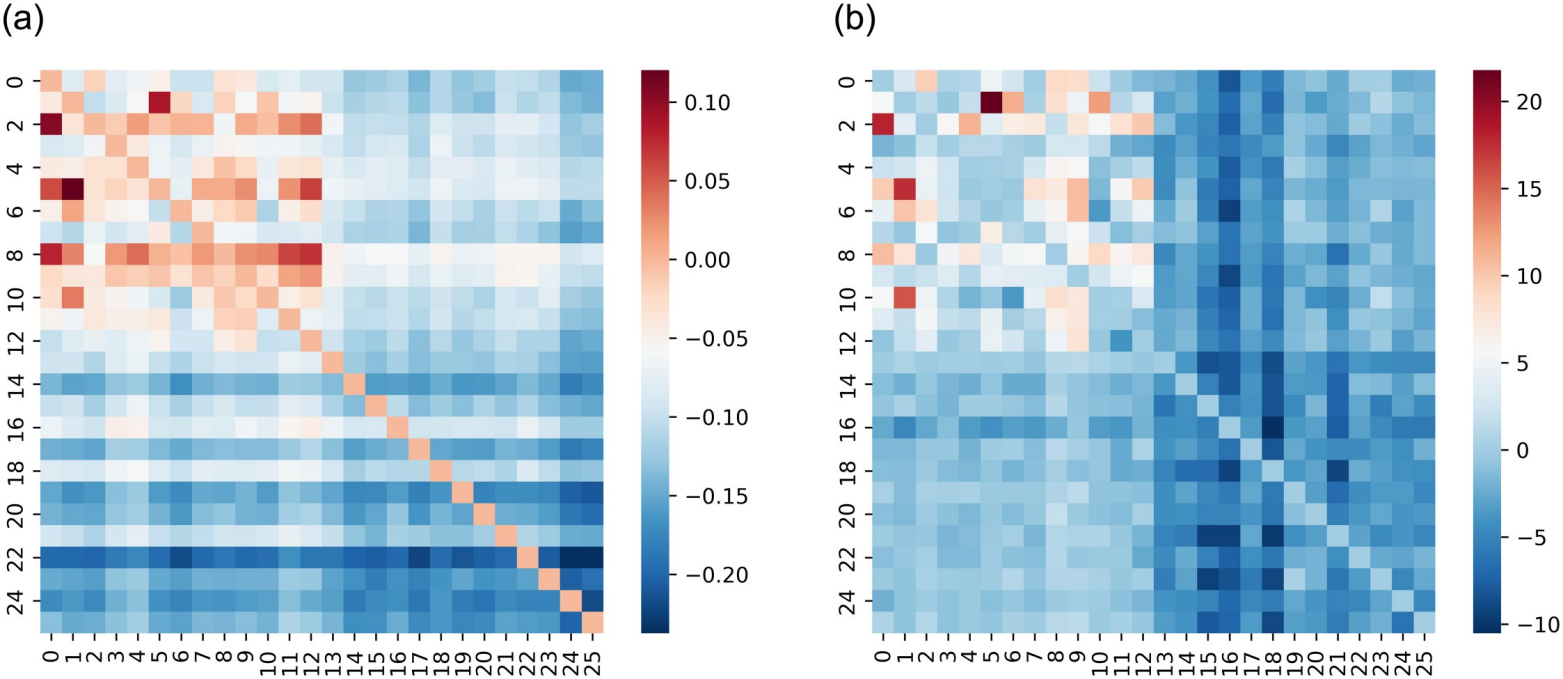
Return&Sent.score—Causality Matrix and Significance (Z-score) Matrix. High information flow among stock Returns. (**a**) TE (Return&Sent.score) (**b**) Significance (Return&Sent.score).

**Fig 7:**
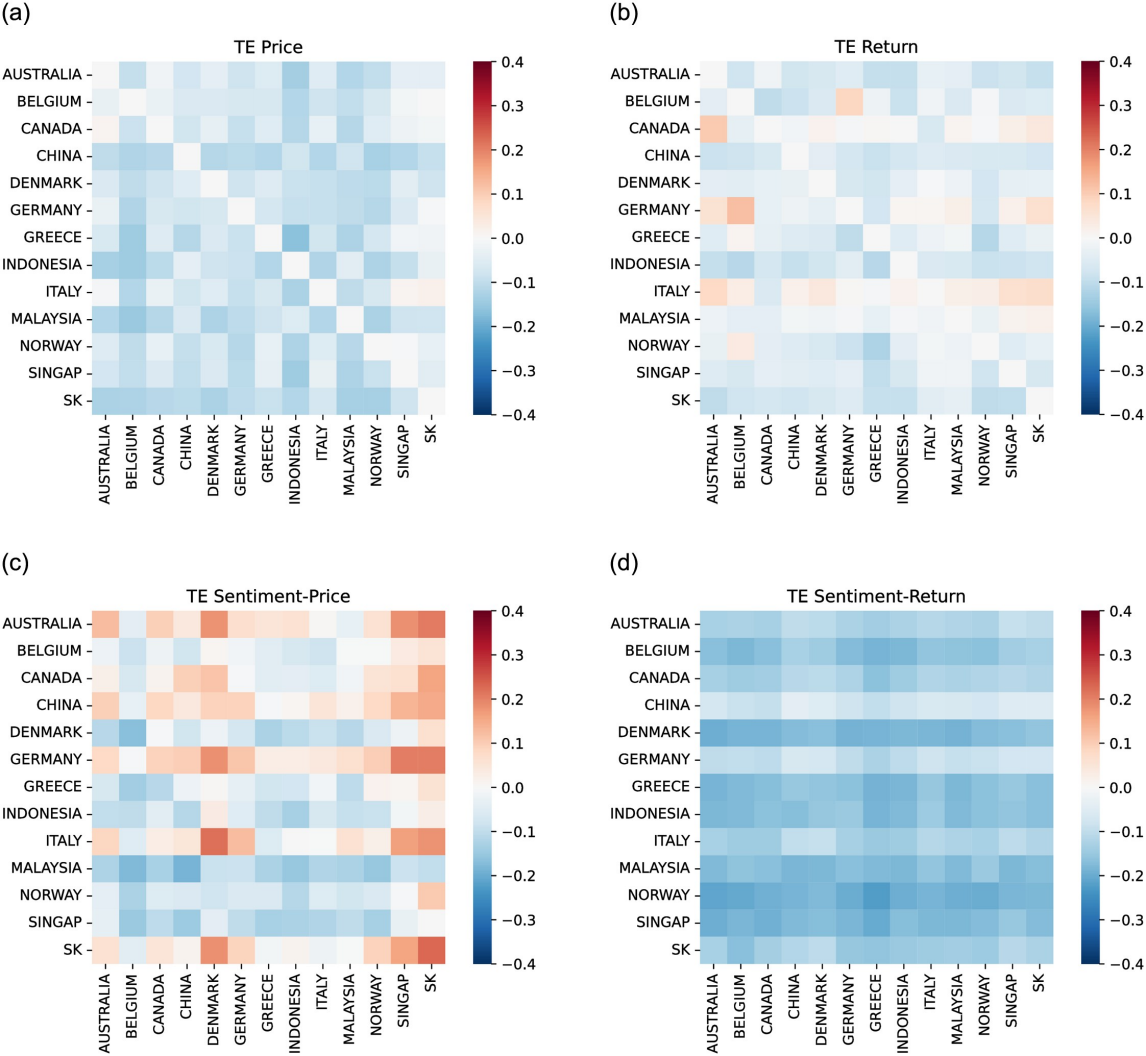
Transfer Entropy between countries. Between Price and Return (top panels) and between Sentiment Index and Price or Return (bottom panels).

For the configuration, Return&Sentiment Score, Fig 6 shows high information flow among stock Returns, as indicated on the top left parts of the matrices. This indicates a strong causality between the stock Returns of the 13 countries. The heat maps also indicate no causal relationships between Sentiment Scores and stock Return. Finally, Fig 7, illustrates the obtained heat maps for the configurations Price&Sentiment Index and Return&Sentiment Index.

TE between the Sentiment Index and Domestic Price is large for many country pairs (see bottom-left panel of Fig 7). It is worth noting that, for Denmark, Singapore, and South Korea, the stock Prices are influenced not only by the news concerning them but also by news related to other countries (see bottom-left panel of Fig 7). The stock Prices of other countries, like Belgium, Indonesia, and Norway, are not influenced by the news concerning them or related to other countries.

From the above analysis, we have found that the combinations Price&Sent.index and Return&Sent.index reveal the most useful information flow. We hypothesize that the information on the TE or Foreign Sentiment Index is expected to be more useful for predicting future domestic Prices than the information on the domestic Sentiment Index or foreign Price. Furthermore, because Domestic Return is not affected by Foreign Sentiment, a different behaviour is expected for Domestic Return than for Domestic Price forecasting.

### 4.2 Dynamic transfer entropy analysis

As introduced in section 2: Related works, Yao [16] extended the use of the Effective Transfer Entropy and Z-score by proposing the Dynamic Transfer Entropy based on a sliding window, and via forward scrolling, the ETE at each time point can be obtained. Yao [16] argued that the lag order found using Transfer Entropy is global and unique and thus unsuitable for capturing the accurate order between two non-stationary series for which the data structure changes, and hence the causal relationships also evolve dynamically.

**Fig 8:**
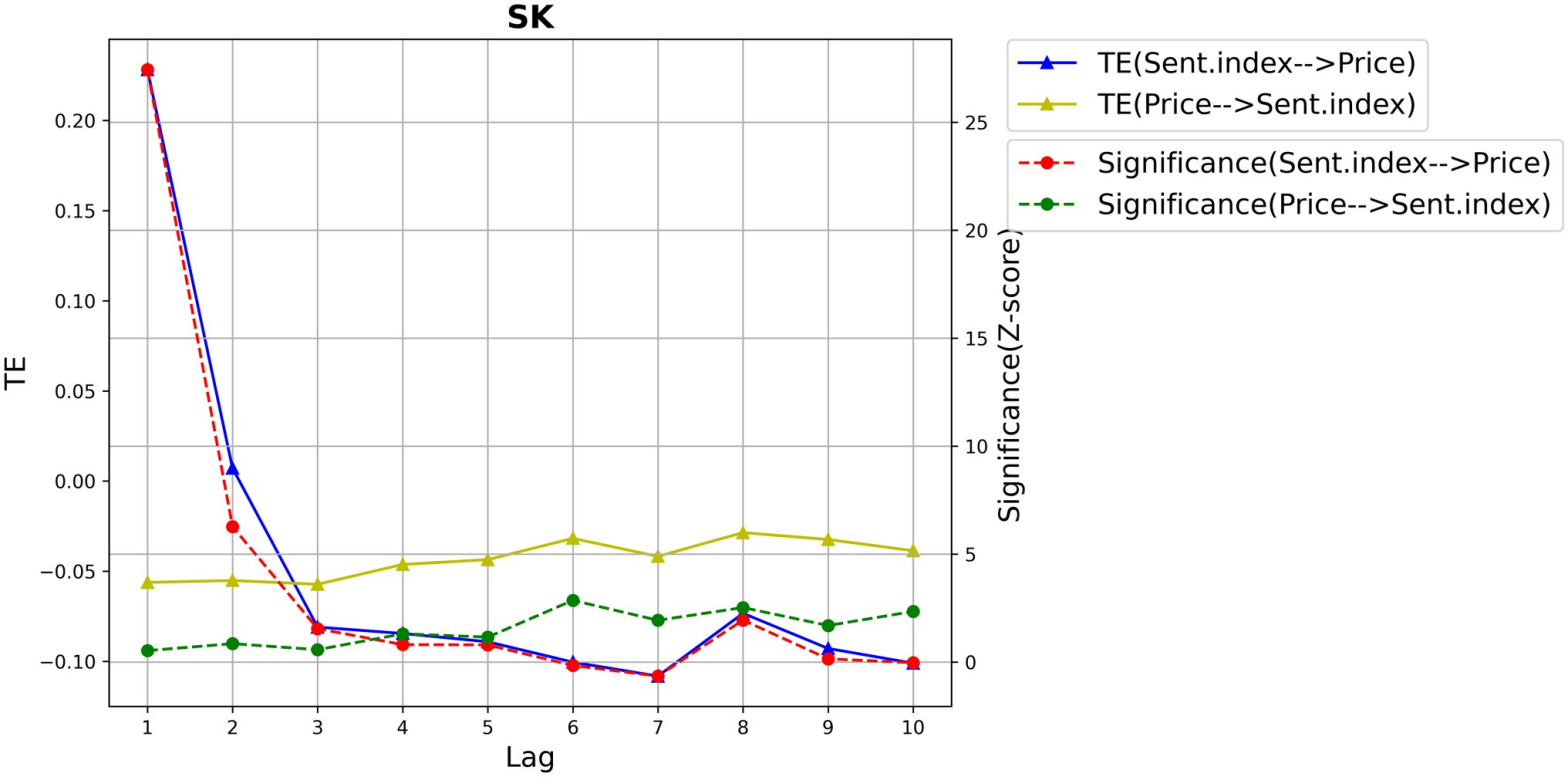
The Dynamic Transfer Entropy identifies the true lag value k = 1 for the case of South Korea. The left y-axis represents TE, and the right y-axis the Significance (Z-score).

We first verified the above claim and performed the information-theoretic analysis to detect the Effective Transfer Entropy at time lags from *k* = 1 up to *k* = 30, and sliding window size = 10. We illustrate in Fig 8 the obtained results for the case of South Korea. The lag *k* = 1 leads to the highest Transfer Entropy value with a Z-score above a significant level (3). As can be seen, the information flows from the Sentiment Index to the stock Price fluctuate considerably (the red and the blue lines), and that flows from the stock Price to the Sentiment Index fluctuate smoothly (the red and the green lines).

**Fig 9:**
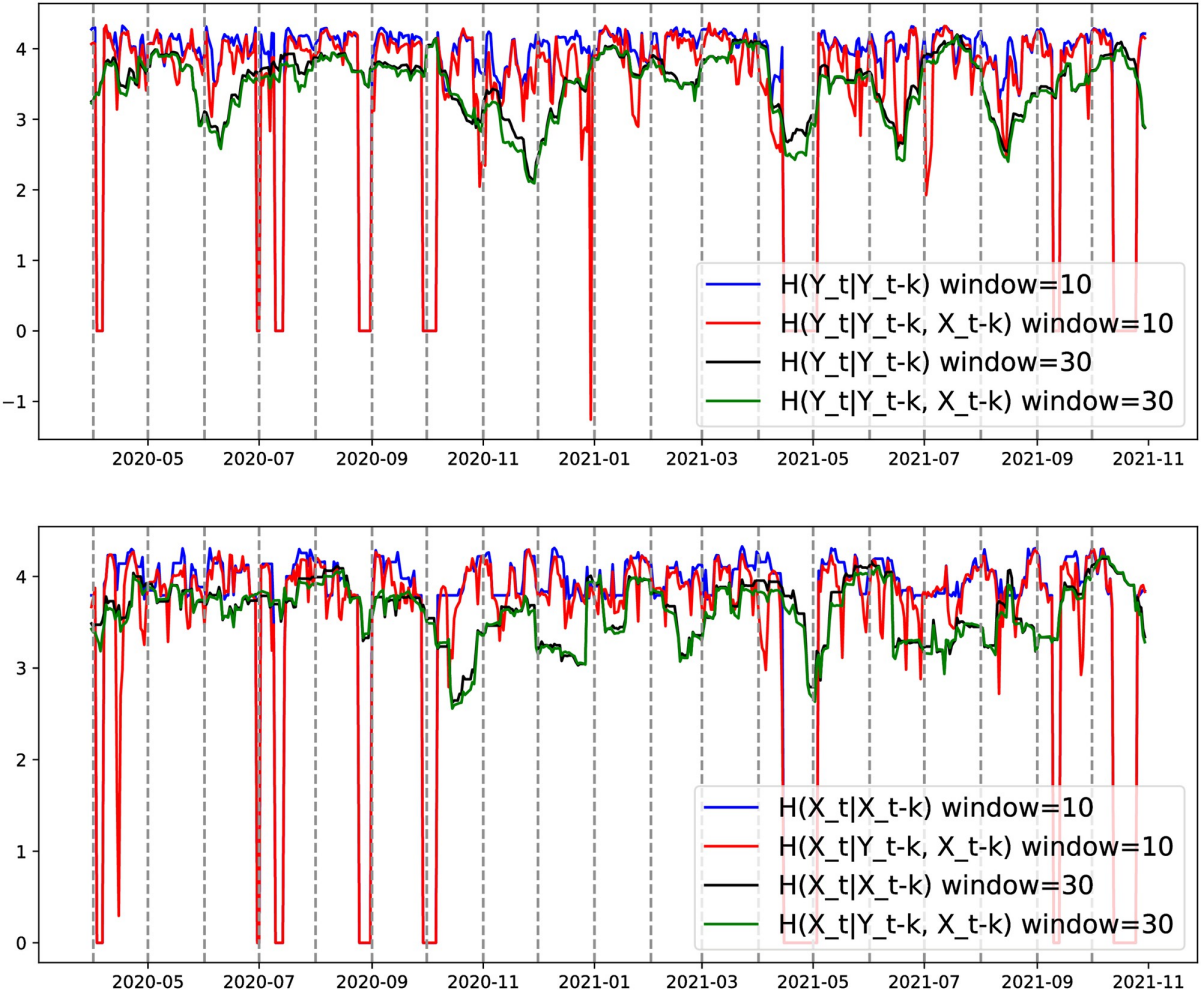
Australia case. Sliding window size effect on the two terms of Eq (5) for DTE estimation. *X* represents the sentiment index time series, and *Y* the stock price time series. The upper panel represents *TE*_*Sent*.*Index*→*Stock*_ and lower *TE*_*Stock*→*Sent*.*Index*_.

The Dynamic Transfer Entropy seeks to extract a collection of time series that reveals the pathway of information transmission between financial news sentiments and stock price fluctuations. This analysis allows us to pinpoint the precise periods when causal changes occur between these two factors. Choosing an appropriate sliding window for estimating DTE is crucial in identifying these specific causal shifts between financial stocks and sentiments. When the window length is large, the two terms in Eq (5) will closely align, resulting in a minimal difference between them as illustrated by the black and green curves of Fig 9. In contrast, when the window is small, we observe distinct differences between the two terms in Eq (5), highlighting shifts in causality between financial stocks and sentiments.

**Fig 10:**
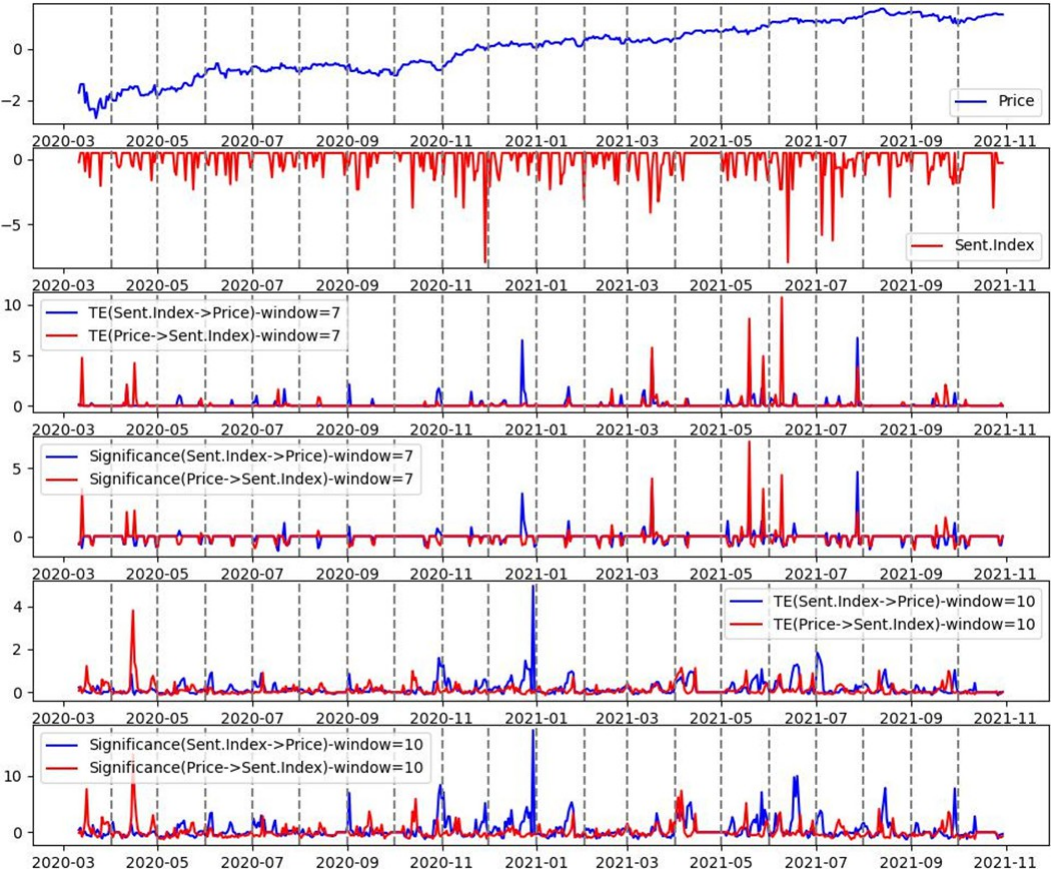
Australia—DTE between stock Price and news sentiment, for the period March 1, 2020, to October 31, 2021. Sliding window size of 7 and 10, with a 1-day step size.

After analyzing several sliding window sizes, we selected a sliding window size of 10 for our experiments and compared it with a sliding window size of 7. In Fig 10, we illustrate the obtained time series for Australia.

As shown from Fig 10, the Dynamic Transfer Entropy provides a dynamic order in the correlation between stock Price and news sentiment. The obtained time series reveals the information transmission path among financial news sentiments and stock Price fluctuations and traces the specific period of causal changes between financial stocks and sentiments, highlighting marginal events such as spikes or sudden jumps, which are crucial in financial time series. As can be seen during specific periods, the stock Price Transfer Entropy to news sentiment is significantly higher than the impact of news sentiment on stock Price and vice-versa for other periods. Additionally, our analysis revealed interesting patterns regarding the Sentiment Indexes, stock Prices, and Transfer Entropy values. Despite the presence of exaggerated negative sentiment in the Sentiment Indexes, we observed different trends between the values of the Sentiment Indexes and the Transfer Entropy (or Z-score) values. Specifically, during periods characterised by exaggerated negative sentiment (such as December 2020, March 2021, June and July 2021), the Transfer Entropy and Z-score values were relatively low.

### 4.3 Stock market forecasting implementation and training details

**Fig 11:**
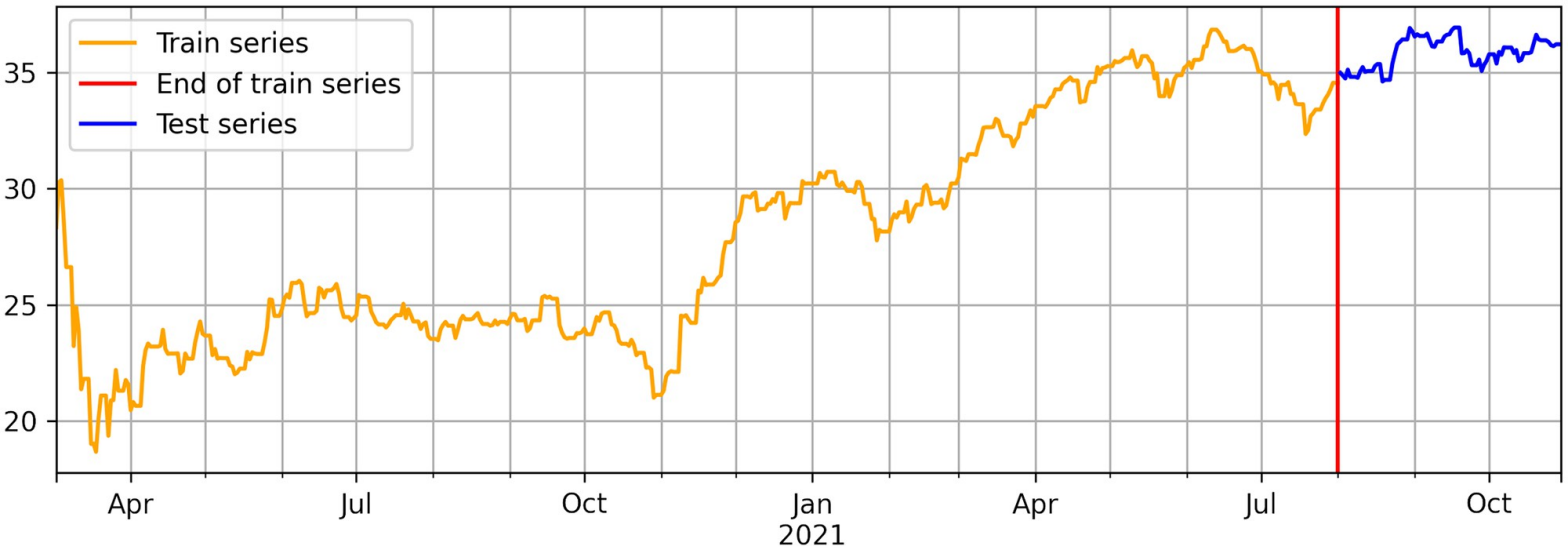
Univariate time series representing the Price for a randomly selected country. The vertical red line illustrates the cutoff time point of the used data for training and testing.

To validate our proposal, we employed the publicly available implementation [65, 66] of the above-described computational methods. We used the Adam optimizer for 20 epochs with a learning rate of 1*e* − 3 and weight decay equal to 1*e* − 8 to train the five considered computational methods. Our objective is a multi-horizon country stock Price or Return, *x*^*i*^, *i* ∈ [0, 12], forecasting. The collected data from DJIA and NYT financial news articles (see section 3.1: Datasets extraction) cover the period from March 02, 2020, to October 31, 2021. We split it into training and testing on August 01, 2021, as illustrated in Fig 11. For the rolling windows employed during training, we used a windows size (i.e., *w*) of 7 and increment (i.e., *m*) of 1 with a multi-horizon prediction length of 7 days. During testing, we directly forecast the upcoming week corresponding to each observed week.

For each of the considered computational methods, we trained 13 models, one for each country. In both training and forecasting, we considered different scenarios to include the co-occurring or associated time-varying covariates (i.e., *V*). Unless otherwise specified, for every scenario involving *TE* as a time-varying covariate, it corresponds to the Dynamic Transfer Entropy estimation employing a window size of 10 days (see section 4.2: Dynamic transfer entropy analysis). The different scenarios are summarised hereafter:

A: No covariates, only the domestic (country) stock (Price or Return), *x*^*i*^, of the country *i*;
B: The domestic stock (Price or Return), *x*^*i*^, of the country *i*, and the country Sent.Index time series as a covariate;
C: The domestic stock (Price or Return), *x*^*i*^, of the country *i*, and the Dynamic Transfer Entropy *TE*_*Sent*.*Index*→*Stock*_ time series as a covariate;
D: The domestic stock (Price or Return), *x*^*i*^, of the country *i*, and the Dynamic Transfer Entropy *TE*_*Stock*→*Sent*.*Index*_ time series as a covariate;
E: The domestic stock (Price or Return), *x*^*i*^, of the country *i*, and the Dynamic Transfer Entropy *TE*_*Sent*.*Index*→*Stock*_ and *TE*_*Stock*→*Sent*.*Index*_ time series as covariates;
F: The domestic stock (Price or Return), *x*^*i*^, of the country *i*, along with the other stock data, i.e. Open Price, Low, High, and the country Senti.Index, the Dynamic Transfer Entropy *TE*_*Sent*.*Index*→*Stock*_ and *TE*_*Stock*→*Sent*.*Index*_ time series as covariates;
G: The domestic stock (Price or Return), *x*^*i*^, of the country *i*, and as covariates the country Sent.Index & Foreign (other countries) Price and their Sent.Index time series;
H: The domestic stock (Price or Return), *x*^*i*^, of the country *i*, and as covariates the country Sent.Index & Foreign Sent.Index time series;
I: The domestic stock (Price or Return), *x*^*i*^, of the country *i*, and as covariates, the foreign (other countries) stocks (Price or Return) time series.

### 4.4 Price and return forecasting

To analyse the performance of TFT and the state-of-the-art computational methods evaluated in this work, we computed the errors between stock predictions yielded by these methods and actual stock values employing three different error measures. Specifically, the error measures used are the root-mean-square error (RMSE), the mean absolute error (MAE), and the symmetric mean absolute percentage error (sMAPE). The formal equations to compute these error measures are as follows [67]:

$$\begin{array}{c} \text{RMSE}=\sqrt{\frac{\sum_{t=1}^{n}{\left({\hat{x}}_{t}^{i}-x_{t}^{i}\right)}^{2}}{n}} \end{array}$$ (30)

$$\begin{array}{c} \text{MAE}=\frac{1}{n}\sum_{t=1}^{n}\left|{\hat{x}}_{t}^{i}-x_{t}^{i}\right| \end{array}$$ (31)

$$\begin{array}{c} \text{sMAPE}=\frac{200}{n}\sum_{t=1}^{n}\frac{|{\hat{x}}_{t}^{i}-x_{t}^{i}|}{\left({\hat{x}}_{t}^{i}+x_{t}^{i}\right)} \end{array}$$ (32)

where ${\hat{x}}_{t}^{i}$ and $x_{t}^{i}$ represent the predicted and actual stock value for the country *i* at time point *t*, and *n* denotes the length of the multi-horizon stock forecasting.

**Fig 12:**
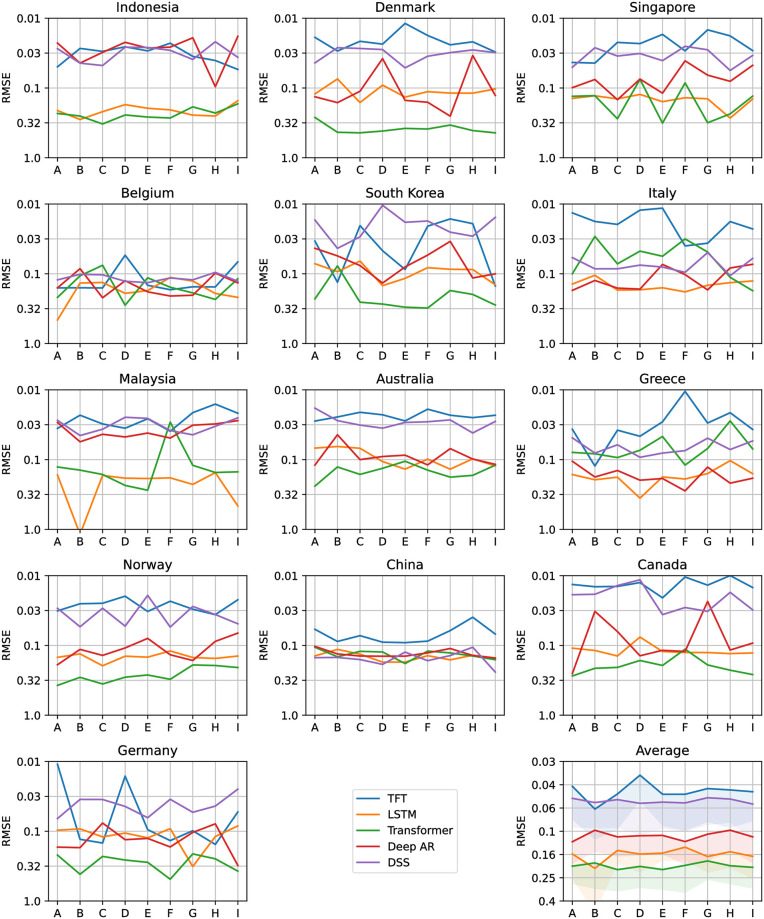
Results corresponding to our Price forecasting using five computational methods: Temporal Fusion Transformers (TFT) [12], Long Short-Term Memory Networks (LSTM) [60], Transformer [11], Probabilistic Forecasting with Autoregressive Recurrent Networks (Deep AR) [54] and Deep State Space (DSS) [53]. The y-axes are in log scale and inverted for better visualization. Each panel shows the Root Mean Squared Error (RMSE) achieved using different variants of covariate inputs from domestic and foreign markets for different countries. The bottom right panel shows the average RMSE across countries with a shaded area denoting average RMSE plus one standard deviation. The different variants of covariates inputs shown on the x-axes are as defined before in section 4.3: Stock market forecasting implementation and training details.

**Fig 13:**
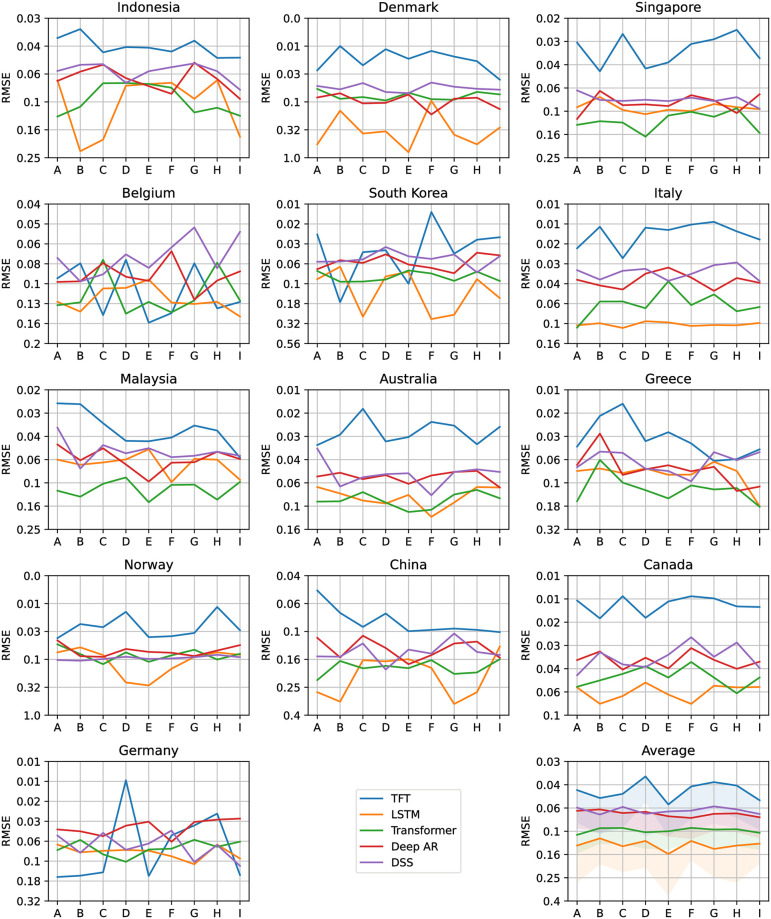
Results corresponding to our Return forecasting using five computational methods: Temporal Fusion Transformers (TFT) [12], Long Short-Term Memory Networks (LSTM) [60], Transformer [11], Probabilistic Forecasting with Autoregressive Recurrent Networks (Deep AR) [54] and Deep State Space (DSS) [53]. The y-axes are inverted for better visualization. Each panel shows the Root Mean Squared Error (RMSE) achieved using different variants of covariate inputs from domestic and foreign markets for different countries. The bottom right panel shows the average RMSE across countries with a shaded area denoting average RMSE plus one standard deviation. The different variants of covariates inputs shown on the x-axes are as defined earlier in section 4.3: Stock market forecasting implementation and training details.

**Fig 14:**
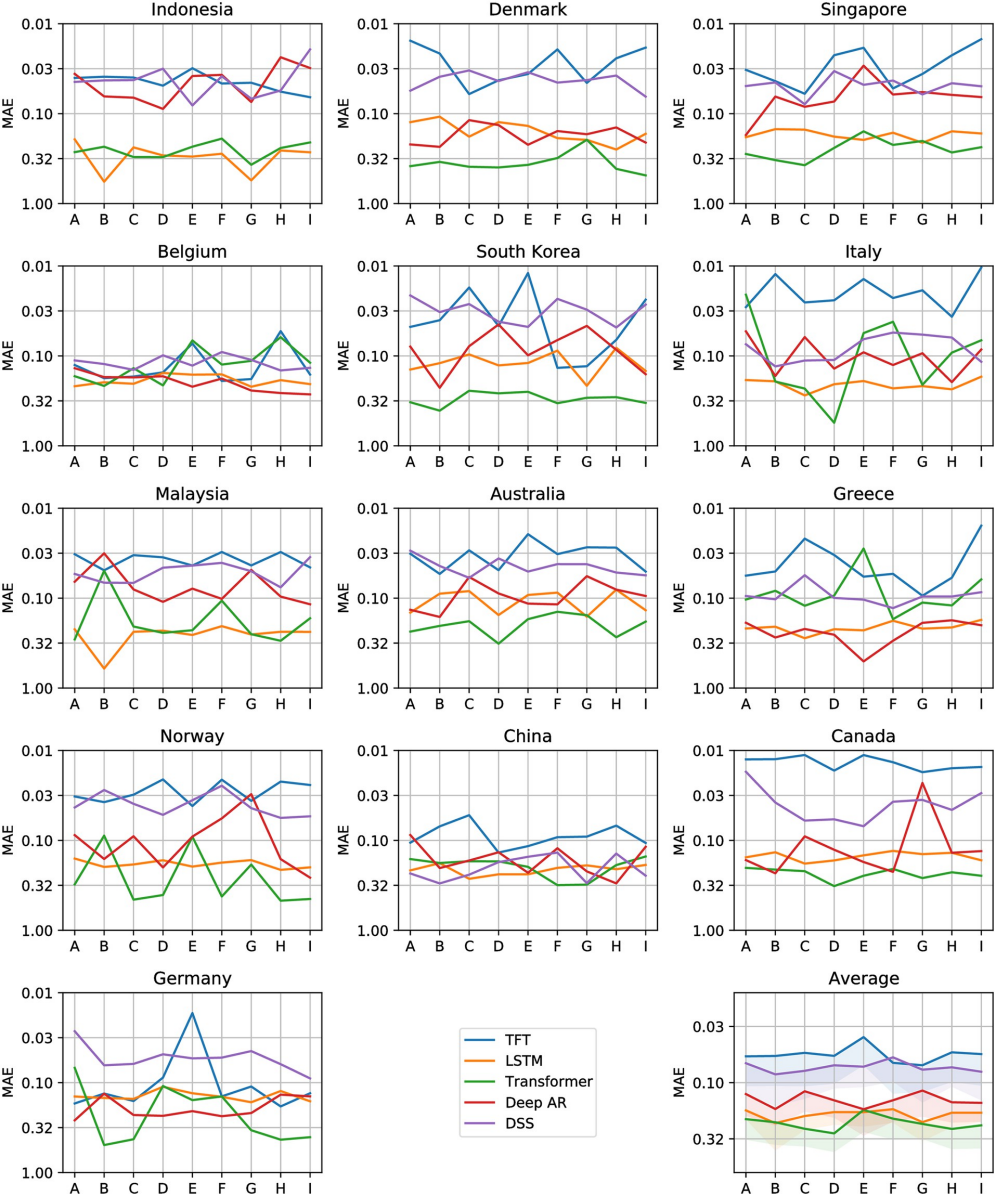
Results corresponding to our Price forecasting using five computational methods: Temporal Fusion Transformers (TFT) [12], Long Short-Term Memory Networks (LSTM) [60], Transformer [11], Probabilistic Forecasting with Autoregressive Recurrent Networks (Deep AR) [54] and Deep State Space (DSS) [53]. The y-axes are in log scale and inverted for better visualization. Each panel shows the Mean Absolute Error (MAE) achieved using different variants of covariate inputs from domestic and foreign markets for different countries. The bottom right panel shows the average MAE across countries with a shaded area denoting average MAE plus one standard deviation. The different variants of covariates inputs shown on the x-axes are as defined before in section 4.3: Stock market forecasting implementation and training details.

**Fig 15:**
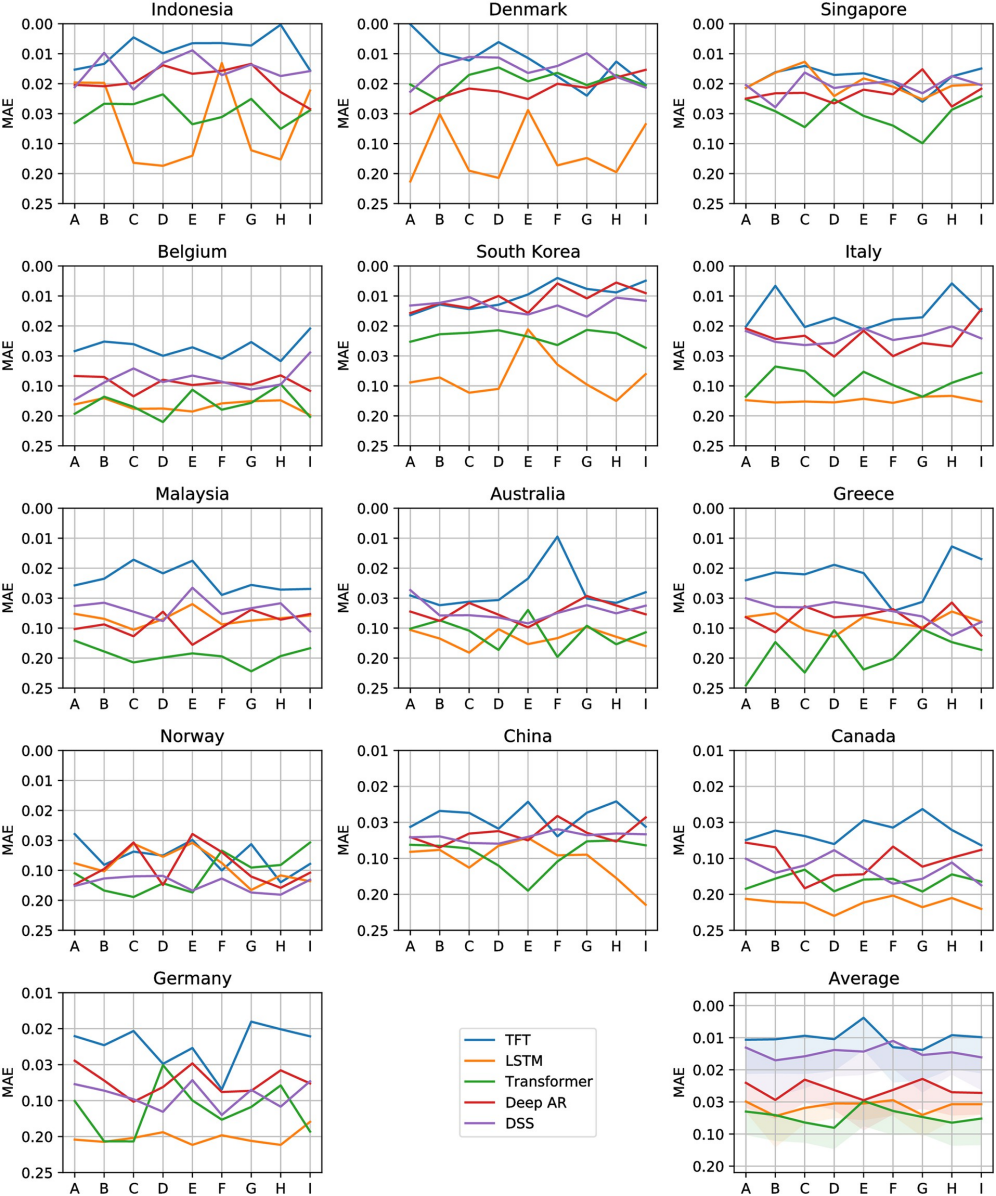
Results corresponding to our Return forecasting using five computational methods: Temporal Fusion Transformers (TFT) [12], Long Short-Term Memory Networks (LSTM) [60], Transformer [11], Probabilistic Forecasting with Autoregressive Recurrent Networks (Deep AR) [54] and Deep State Space (DSS) [53]. The y-axes are inverted for better visualization. Each panel shows the Mean Absolute Error (MAE) achieved using different variants of covariate inputs from domestic and foreign markets for different countries. The bottom right panel shows the average MAE across countries with a shaded area denoting average MAE plus one standard deviation. The different variants of covariates inputs shown on the x-axes are as defined earlier in section 4.3: Stock market forecasting implementation and training details.

**Fig 16:**
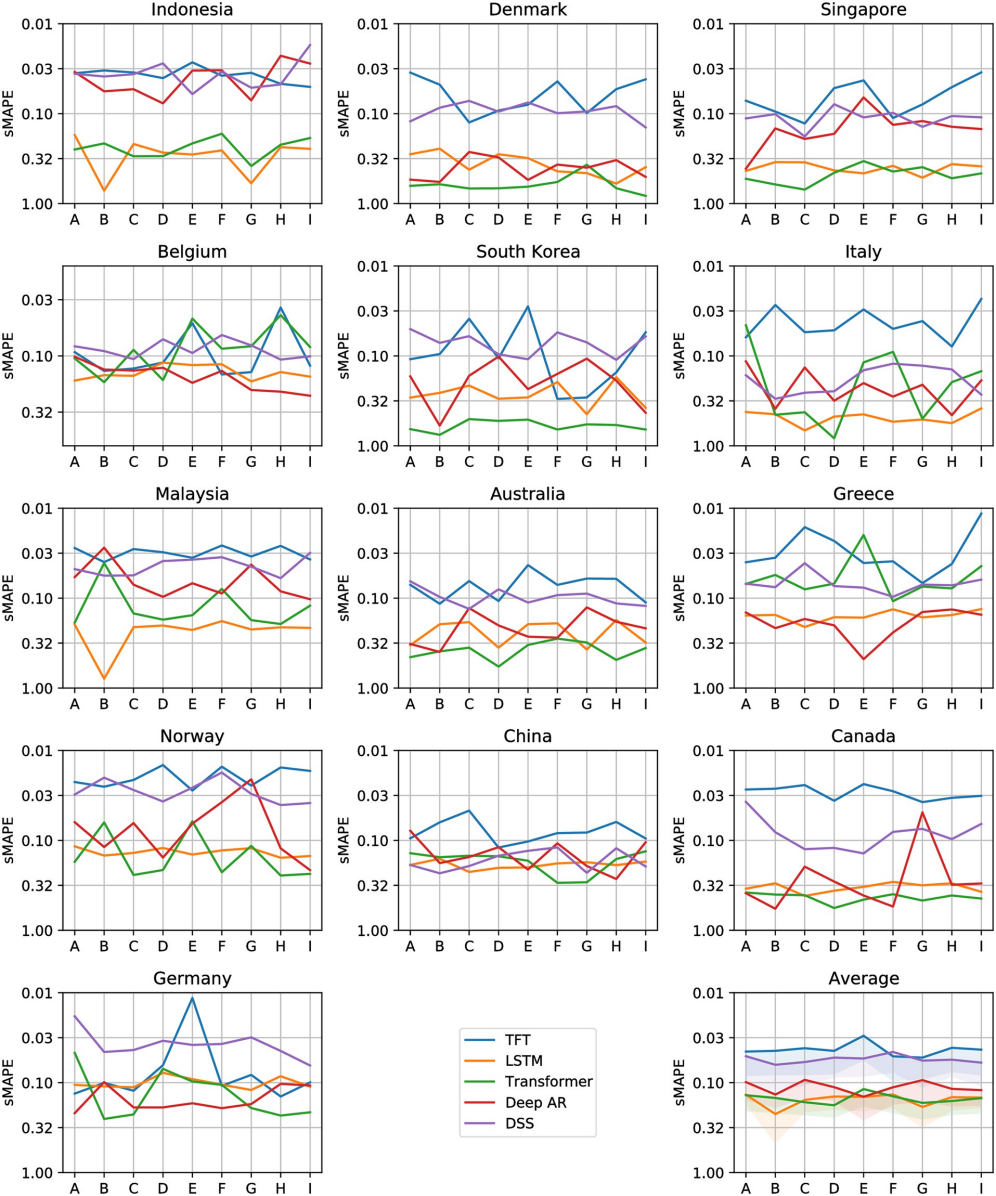
Results corresponding to our Price forecasting using five computational methods: Temporal Fusion Transformers (TFT) [12], Long Short-Term Memory Networks (LSTM) [60], Transformer [11], Probabilistic Forecasting with Autoregressive Recurrent Networks (Deep AR) [54] and Deep State Space (DSS) [53]. The y-axes are in log scale and inverted for better visualization. Each panel shows the Symmetric Mean Absolute Percentage Error (sMAPE) achieved using different variants of covariate inputs from domestic and foreign markets for different countries. The bottom right panel shows the average sMAPE across countries with a shaded area denoting average sMAPE plus one standard deviation. The different variants of covariates inputs shown on the x-axes are as defined before in section 4.3: Stock market forecasting implementation and training details.

**Fig 17:**
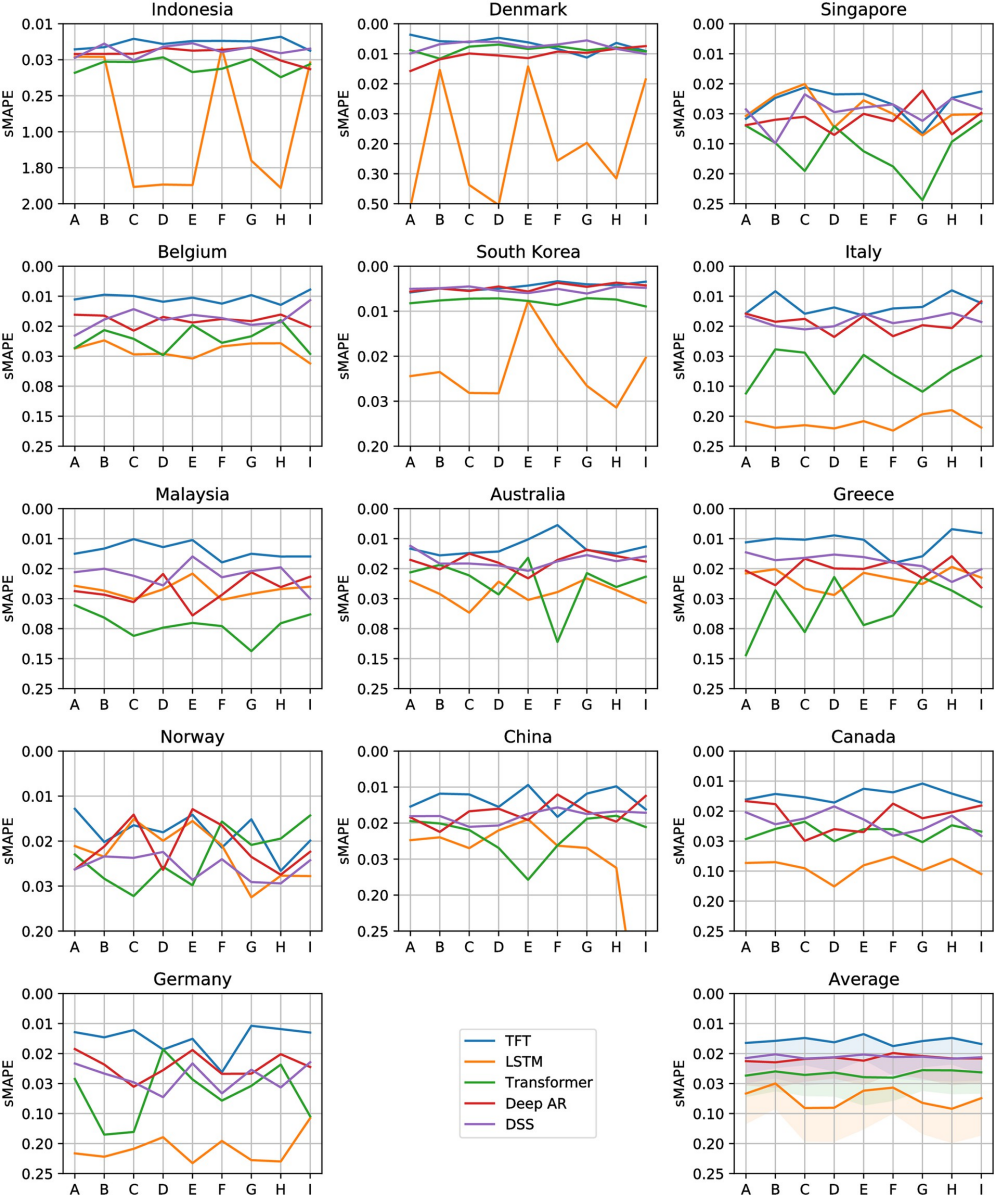
Results corresponding to our Return forecasting using five computational methods: Temporal Fusion Transformers (TFT) [12], Long Short-Term Memory Networks (LSTM) [60], Transformer [11], Probabilistic Forecasting with Autoregressive Recurrent Networks (Deep AR) [54] and Deep State Space (DSS) [53]. The y-axes are inverted for better visualization. Each panel shows the Symmetric Mean Absolute Percentage Error (sMAPE) achieved using different variants of covariate inputs from domestic and foreign markets for different countries. The bottom right panel shows the average sMAPE across countries with a shaded area denoting average sMAPE plus one standard deviation. The different variants of covariates inputs shown on the x-axes are as defined earlier in section 4.3: Stock market forecasting implementation and training details.

Figs 12–17 show the performance of the five computational methods for stock forecasting. Figs 12 and 13 summarize the performance of the methods for Price and Return forecasting, respectively, measured in terms of RMSE. Figs 14 and 15 illustrate Price and Return forecasting performance measured using MAE. Figs 16 and 17 present the methods’ performance corresponding to Price and Return forecasting measured with sMAPE.

From Figs 12 to 17 each panel shows the forecasting errors for a different country except for the bottom left panel, which shows the average across all countries. The y-axis is log-scaled and inverted for better visualization. Therefore, the highest-performing models are above the lowest-performing models. To account for the performance variability across countries of each model, shaded areas are added to the model averages (bottom left panel), indicating the average error plus one standard deviation. The x-axis of each panel denotes the different variants of covariates used to train the models (see the details in section 4.3: Stock market forecasting implementation and training details). In general, from Figs 12 to 17, one can notice that the highest-performing computational method is the TFT. These results confirm the effectiveness of TFT in delivering promising outcomes for stock forecasting. The success of TFT stems from the integration of Variable Selection and Gated Residual Networks in conjunction with the Interpretable Attention blocks, which lead to a combination of attention, feature selection, and interpretation mechanisms, resulting in a robust computational method suitable for stock movement forecasting. In the following, we analyse the behaviour of TFT for different variants of input covariates. Finally, we discuss its benefits in providing actionable insights into the importance of the time-varying covariates for interpretable stock forecasting.

### 4.5 Effect of covariates on TFT model

**Fig 18:**
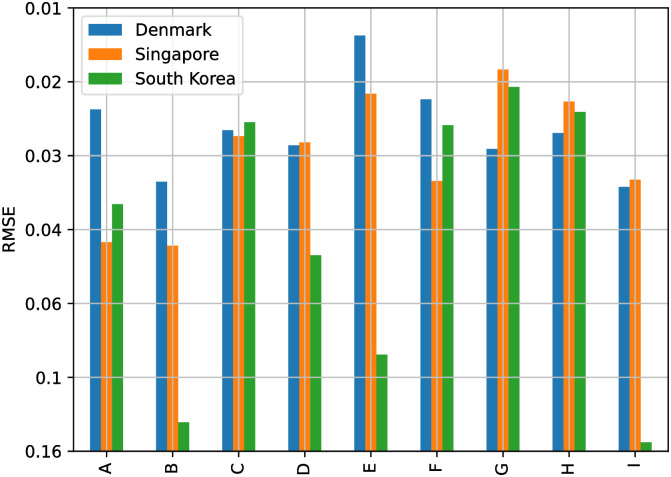
Price forecasting error in Denmark, Singapore, and South Korea for the highest-performing computational method, TFT. These three countries present the largest Dynamic Transfer Entropy from Sentiment to Price. It can be observed that the variants of covariates (see section 4.3: Stock market forecasting implementation and training details.), including only Dynamic Transfer Entropy terms (C, D, and E) or foreign Sentiment terms (F, G, and H), have lower errors than the variants including only domestic Sentiment (B) or only domestic Price (I) in addition to domestic Price.

To illustrate the potential utility of Dynamic Transfer Entropy analysis on model design, Fig 18 shows the Price forecasting performance in three countries, in Denmark, Singapore and South Korea, with the largest effect of foreign Sentiment Index on domestic Price. For the three countries, the variants of time-varying covariates (see section 4.3: Stock market forecasting implementation and training details), including the domestic Price, the Dynamic Transfer Entropy information (C, D and E) or foreign Sentiment Index (F, G and H) have lower errors than the variants including only domestic Sentiment Index (B) or only foreign Price (I). This confirms the Transfer Entropy analysis of the Causality Matrix in Fig 7, which illustrates that foreign Sentiment Index, not foreign Price, predicts future domestic Price (top and bottom right panels).

**Fig 19:**
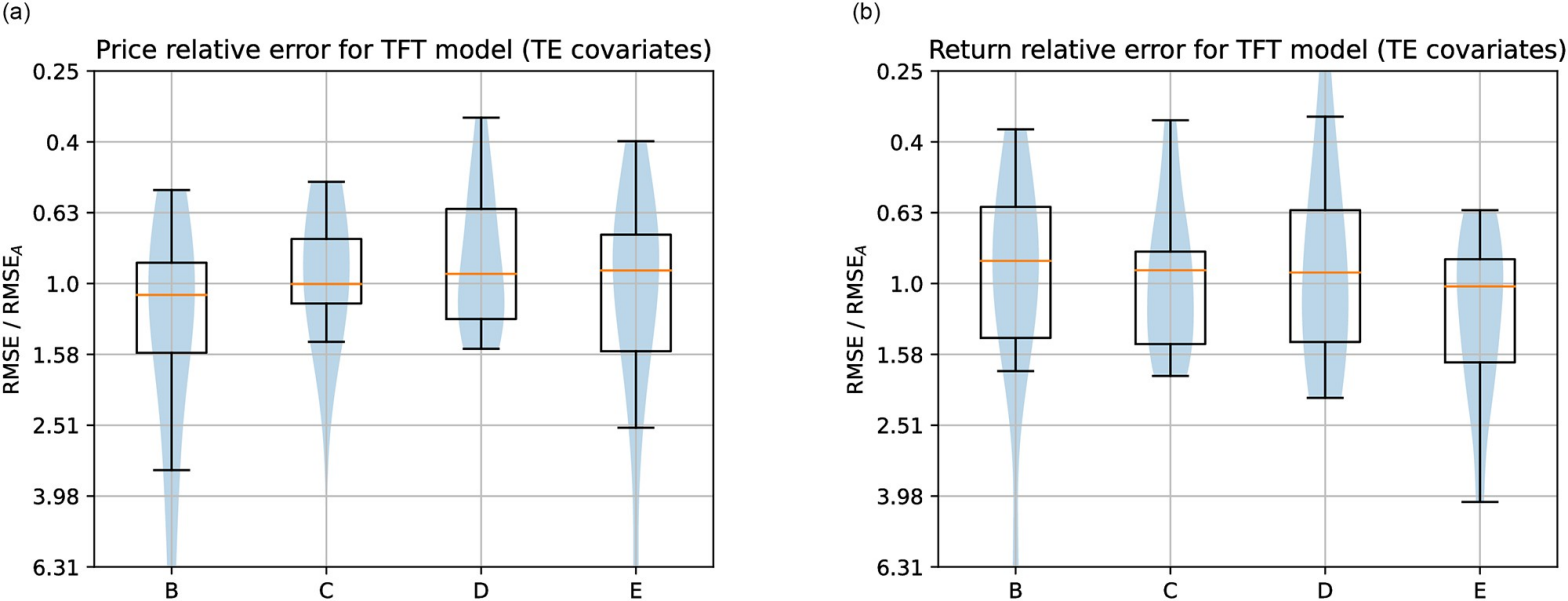
**Left: Summary of Price forecasting error distributions relative to the variant of covariates A (only domestic Price) for the highest-performing computational method, TFT, and for the variants of covariates B, C, D, and E**. All the variants, including Transfer Entropy information, obtained a median error smaller than the variant using only the Sentiment Index in addition to domestic Price, and the variants D and E obtained lower errors than using only domestic Price. **Right: Summary of Return forecasting error distributions relative to variant of covariates A**. Contrary to Price forecasting, Dynamic Transfer Entropy terms did not improve over Sentiment Index.

**Fig 20:**
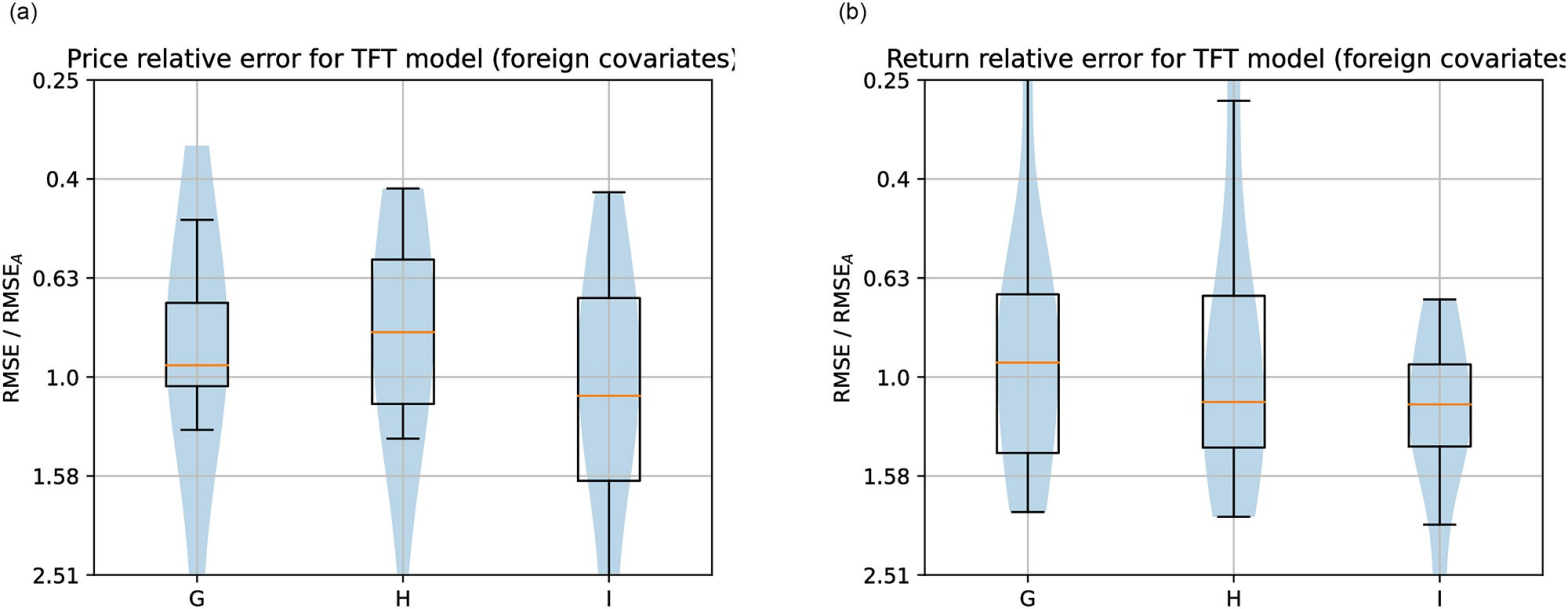
**Left: Summary of Price forecasting error distributions relative to A variant of covariates (only domestic Price) for the variants that include foreign Sentiment Index (G and H) or only Foreign Price (I), in addition to domestic Price**. The variants, including the foreign Sentiment Index, obtained a median error smaller than the reference (A: domestic Price) and than the variant using only foreign Price in addition to domestic Price. **Right: Summary of Return forecasting error distributions relative to variant A of covariates**. Variant H (Sentiment Index and foreign Sentiment Index) did not improve over variant A, domestic Price.

**Fig 21:**
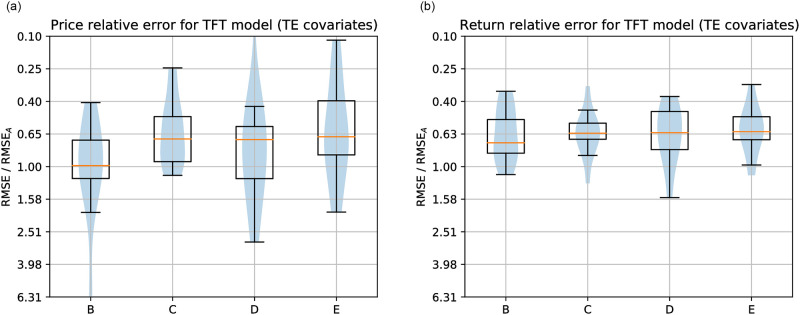
**Left: Summary of Price forecasting error distributions relative to the variant of covariates A (only domestic Price) for the highest-performing computational method, TFT, and for the variants of covariates B, C, D and E**. For all the variants in this illustration, the Dynamic Transfer Entropy was estimated using a window size equal to 7 days. All variants obtained a median error smaller than the variant using only the Sentiment Index in addition to domestic Price, and variants D and E obtained lower errors than using only domestic Price. **Right: Summary of Return forecasting error distributions relative to variant of covariates A**. Similarly to Price forecasting, Dynamic Transfer Entropy terms also improve over Sentiment Index as all variants obtained a median error smaller than the variant using only the Sentiment Index.

Figs 19–21 show the TFT model performances for Price and Return forecasting using different variants of input covariate relative to variant A, which uses only domestic Price or Return, respectively. The boxplots show the median and interquartile range (Q3-Q1) of the RMSE for each variant divided by the RMSE of variant A across countries.

Fig 19 illustrates the effect of adding Dynamic Transfer Entropy parameters (variants C, D, and E) compared with the Sentiment Index (variant B) (see section 4.3: Stock market forecasting implementation and training details) For Price forecasting, adding the Sentiment Analysis parameters alone as covariate impairs performance while the Transfer Entropy information improves the prediction (see B distribution on the left panel). The opposite trend is observed for the Return forecasting when the model trained using the Sentiment Index performs best. This behaviour is in agreement with the Dynamic Transfer Entropy analysis shown in Fig 7, which shows that the Sentiment Index of other countries affects the domestic Price (bottom left panel) but not the Return (bottom right panel).

Fig 20 shows the effect of foreign Price or Return on the prediction of Price and Return, respectively. For Price forecasting, the median of the error distribution for variant H (see section 4.3: Stock market forecasting implementation and training details), which includes the foreign Sentiment Index, is higher than for variant I, which includes only foreign Price. Again, this behaviour agrees with the Transfer Entropy analysis shown in Fig 7. The bottom left panel of Fig 7 illustrates that the Sentiment Index of other countries helps in domestic Price forecasting while the foreign Price (top left panel) does not.

Fig 21 portrays the effect of adding Dynamic Transfer Entropy time-varying covariate but estimated using a window size of 7 days (see section 4.2: Dynamic transfer entropy analysis). Similarly to Fig 19, the scenarios using Dynamic Transfer Entropy (variants C, D, and E) are compared with those using Sentiment Index (variant B) (see section 4.3: Stock market forecasting implementation and training details) for Price forecasting. Employing Dynamic Transfer Entropy information computed with a window size of 7 days brings more benefits to enhancing the model’s performance than using only the Sentiment Index. These outcomes suggest that operating with a uniform window size for Dynamic Transfer Entropy estimation and stock market prediction enables capturing the dynamic information flow between time-varying covariates more effectively.

### 4.6 TFT interpretability

**Fig 22:**
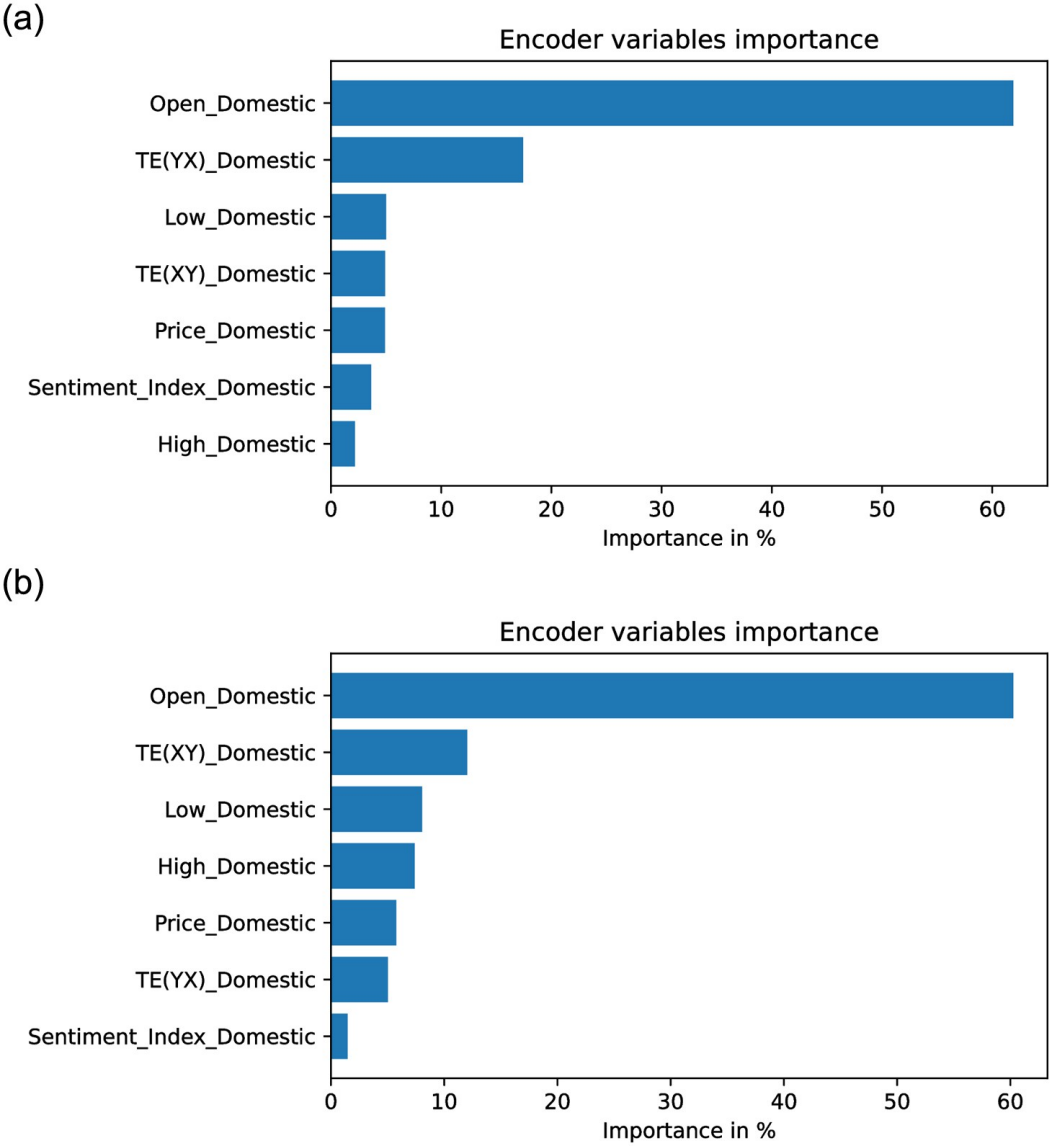
Importance of encoder variables for country stock Price forecasting. The Variable Selection Network of TFT generally selects *TE*_*Stock*→*Sent*.*Index*_ as the second most important variable. (**a**) Singapore (**b**) South Korea.

To confirm the benefits of the Dynamic Transfer Entropy in stock forecasting, we analyzed the variable importance scores for the different inputs used for forecasting. To conduct this analysis, we built upon the TFT open source implementation available at https://pytorch-forecasting.readthedocs.io/en/latest/index.html. As mentioned above, in this study, we considered only covariates for which past values are known at prediction time, hence we analysed only the variables’ importance at the TFT encoder. To confirm the results shown in Fig 18, we examined the importance of the variables considered in variant F, namely, the country Open Price, Low, High, and country Senti.Index, the Dynamic Transfer Entropy *TE*_*Sent*.*Index*→*Stock*_, and *TE*_*Stock*→*Sent*.*Index*_. Our analysis confirmed the importance of Open Price, *TE*_*Sent*.*Index*→*Stock*_ and *TE*_*Stock*→*Sent*.*Index*_ in the prediction of stock Price, summing almost 80% of the weighted importance for all the considered countries. Also, we noticed that for most of the countries, the information flows from Stock to Sent.Index, i.e *TE*_*Stock*→*Sent*.*Index*_, has higher importance (20%), compared to *TE*_*Sent*.*Index*→*Stock*_, as illustrated in the left panel of Fig 22 for Singapore. Only for Italy and South Korea *TE*_*Sent*.*Index*→*Stock*_ had higher importance, as depicted in the right panel of Fig 22 for South Korea.

**Fig 23:**
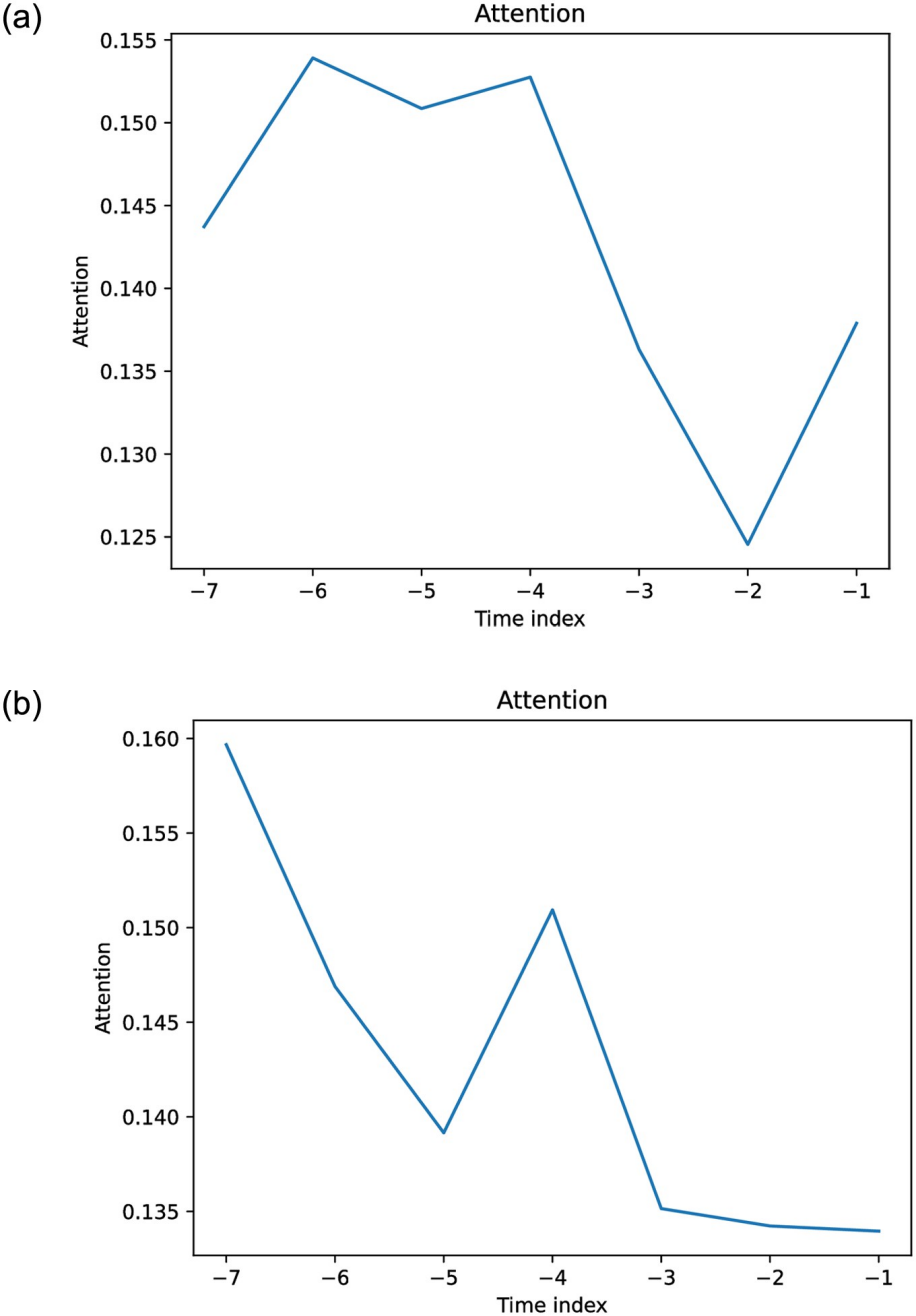
Average attention learned by TFT. The attention weight patterns varies across countries. (**a**) Denmark (**b**) Singapore.

Fig 23 represents the attention weight patterns for one-step-ahead stock Price forecasts, for Denmark and Singapore. The attention weights can be used to understand the most important past-time steps that the TFT model focuses on. The horizontal axis is the relative time index compared to the target time 0. The result suggests that the time step gets a higher weight when the relative time index is further to 0. This means that the model relies mainly on a relatively larger window of days, i.e., approximately the previous 4 to 7 days, for prediction.

**Fig 24:**
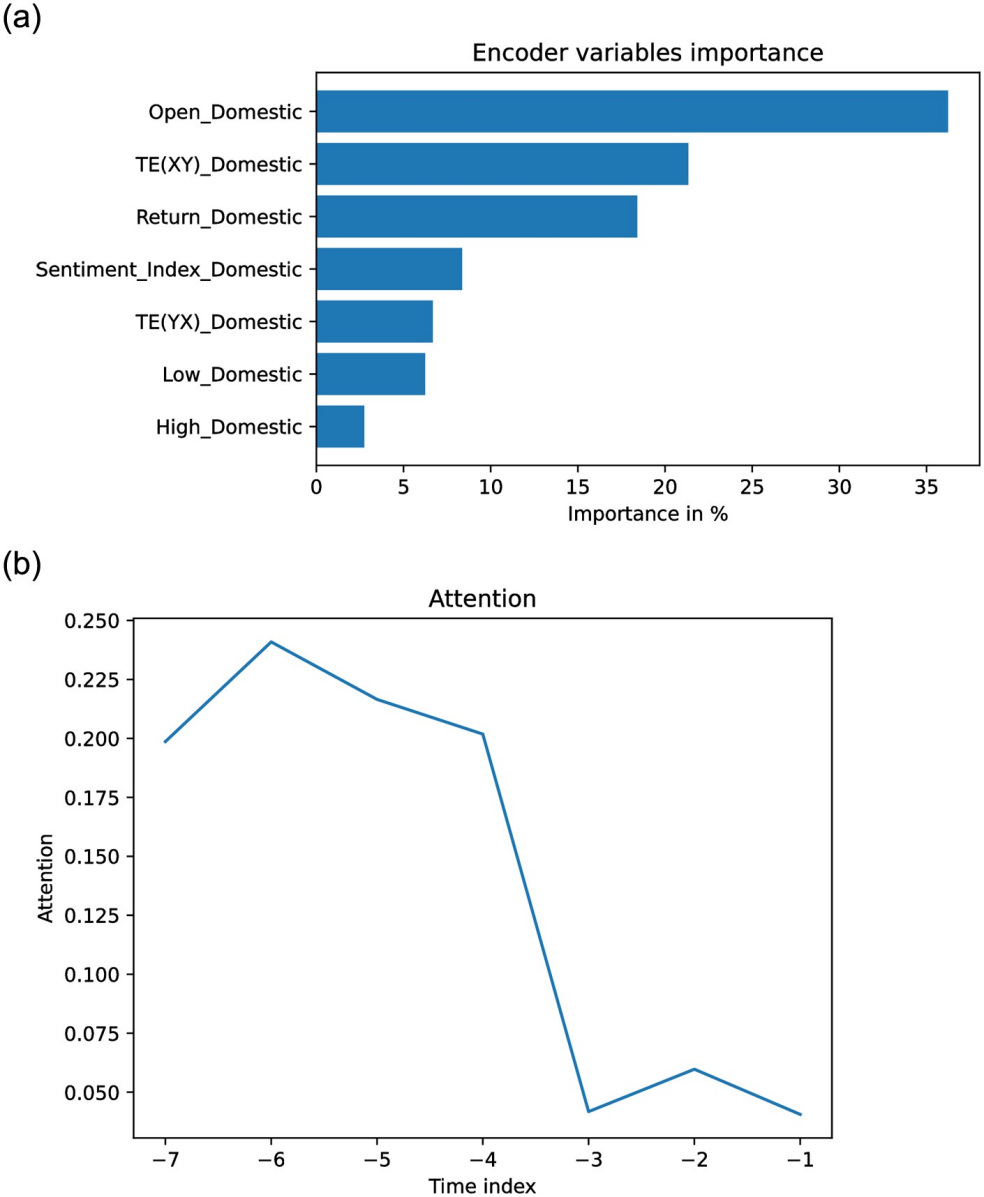
TFT Interpretability for South Korea. Illustrations corresponding to stock Return forecasting. (**a**) Importance of Encoder variables (**b**) Attention weight.

In the case of stock Return forecasting, Open Price, and *TE*_*Sent*.*Index*→*Stock*_ plays an important role in the prediction, summing almost 70% of the weighted importance for all of the considered countries, as illustrated in the left panel of Fig 24. The right panel depicts the attention weights pattern, and as it can be seen the model relies on the previous 6 days for prediction.

## 5 Conclusion

In this study, we proposed using the Temporal Fusion Transformer (TFT) [12] for stock movement prediction, which efficiently correlates multiple factors affecting stock markets. Dynamic Transfer Entropy [16] has been adopted to model causality interactions between stock and news sentiments, which made significant contributions to stock movement prediction, leading to improved prediction accuracy. Five models were compared for stock market prediction, TFT was superior for predicting stock Price/Return with the lowest errors among all models. We showed that TFT is a powerful architecture for stock movement forecasting. Most notably, its self-attention makes it aware of all variables and their dependencies throughout every time step, to pay more attention to certain variables. Moreover, in contrast to other forecasting models, TFT has proven to be effective for multi-horizon forecasting, which makes it useful for stock movement forecasting due to its ability to use time-varying future inputs. Furthermore, by using interpretable attention, TFT can show the importance of the variables that are used to make forecasts.

Our results successfully validated the rationale behind utilizing sentiment as a predictive factor for stock Prices/Returns of specific countries. The proposed methodology demonstrated the existence of meaningful relationships and highlighted the potential utility of sentiment analysis and Dynamic Transfer Entropy in predicting stock market behaviour. The Dynamic Transfer Entropy allowed for obtaining time series that reveals the information transmission path among financial news sentiments and stock Price/Return fluctuations and traces the specific period of causal changes between financial stocks and sentiments. To the best of our knowledge, this is the first study that uses Dynamic Transfer Entropy as exogenous data to predict stock Price/Return. The other contribution of this work is a country-de-biased FinBERT model for sentiment analysis of financial newspapers. For this, we applied fine-tuning to the last layers of the FinBERT model by imposing a fairness constraint.

We used the Transfer Entropy to analyse the correlation between countries’ stock Prices, which showed causality between foreign stocks. However, we only used the foreign stocks as covariates for a fair comparison between the different forecasting models, which in our case did not improve the prediction. In future work, we planned to include the Causality Matrix of the Transfer Entropy between stock Price time series as input to the TFT, making use of all types of inputs allowed by the model, i.e., static, past-observed time-varying input and future-known time-varying input. Moreover, our study considered only stock Price/Return and sentiments in news articles, in the future we planned to follow the new Adaptive Market Hypothesis (AMH) theory [68] by incorporating, on top of stock Price/Return and Sentiment, technical indicators, such as the moving average convergence divergence (MACD) and the relative strength index (RSI) [69], that summarize many aspects of the historical Price and are often used by investors to analyse market states.
